# The Membrane-Bound C Subunit of Reductive Dehalogenases: Topology Analysis and Reconstitution of the FMN-Binding Domain of PceC

**DOI:** 10.3389/fmicb.2018.00755

**Published:** 2018-04-24

**Authors:** Géraldine F. Buttet, Mathilde S. Willemin, Romain Hamelin, Aamani Rupakula, Julien Maillard

**Affiliations:** ^1^Laboratory for Environmental Biotechnology, Institute for Environmental Engineering, Swiss Federal Institute of Technology in Lausanne, Lausanne, Switzerland; ^2^Protein Core Facility, Faculty of Life Sciences, Swiss Federal Institute of Technology in Lausanne, Lausanne, Switzerland

**Keywords:** organohalide respiration, PceC, RdhC, flavoproteins, flavin mononucleotide (FMN), protein reconstitution, flavin-trafficking proteins (Ftp), flavin transferase

## Abstract

Organohalide respiration (OHR) is the energy metabolism of anaerobic bacteria able to use halogenated organic compounds as terminal electron acceptors. While the terminal enzymes in OHR, so-called reductive dehalogenases, are well-characterized, the identity of proteins potentially involved in electron transfer to the terminal enzymes remains elusive. Among the accessory genes identified in OHR gene clusters, the C subunit (*rdhC*) could well code for the missing redox protein between the quinol pool and the reductive dehalogenase, although it was initially proposed to act as transcriptional regulator. RdhC sequences are characterized by the presence of multiple transmembrane segments, a flavin mononucleotide (FMN) binding motif and two conserved CX_3_CP motifs. Based on these features, we propose a curated selection of RdhC proteins identified in general sequence databases. Beside the Firmicutes from which RdhC sequences were initially identified, the identified sequences belong to three additional phyla, the Chloroflexi, the Proteobacteria, and the Bacteriodetes. The diversity of RdhC sequences mostly respects the phylogenetic distribution, suggesting that *rdhC* genes emerged relatively early in the evolution of the OHR metabolism. PceC, the C subunit of the tetrachloroethene (PCE) reductive dehalogenase is encoded by the conserved *pceABCT* gene cluster identified in *Dehalobacter restrictus* PER-K23 and in several strains of *Desulfitobacterium hafniense*. Surfaceome analysis of *D. restrictus* cells confirmed the predicted topology of the FMN-binding domain (FBD) of PceC that is the exocytoplasmic face of the membrane. Starting from inclusion bodies of a recombinant FBD protein, strategies for successful assembly of the FMN cofactor and refolding were achieved with the use of the flavin-trafficking protein from *D. hafniense* TCE1. Mass spectrometry analysis and site-directed mutagenesis of rFBD revealed that threonine-168 of PceC is binding FMN covalently. Our results suggest that PceC, and more generally RdhC proteins, may play a role in electron transfer in the metabolism of OHR.

## Introduction

Organohalide respiration (OHR) is a respiratory metabolism that uses halogenated compounds as terminal electron acceptors, and allows an increasing number of anaerobic bacteria to conserve energy (Adrian and Löffler, 2016). While there is an extensive body of information on the reductive dehalogenases (RdhA, RDases), the key enzymes involved in the catalytic reduction of organohalides (for a review, see Jugder et al., 2016a), relatively little is known about the electron transport in OHR, and specifically about the redox proteins involved in donating electrons to RDases. Nevertheless, models of electron transport have been recently proposed (Goris et al., 2015b; Kublik et al., 2016; Maillard and Holliger, 2016; Fincker and Spormann, 2017), indicating that, depending on their phylogeny, organohalide-respiring bacteria (OHRB) must have developed various strategies to deliver electrons to the corrinoid cofactor of RDases at sufficiently low redox potential. Indeed, the Co^II^/Co^I^ midpoint reduction potential for the corrinoid in the tetrachloroethene RDase (PceA) of *Dehalobacter restrictus* and in the chlorophenol RDase (CprA) of *Desulfitobacterium dehalogenans* have been measured at -350 and -370 mV, respectively (Schumacher et al., 1997; van de Pas et al., 1999). While menaquinones are involved in electron transfer to RDases in *D. restrictus* (Schumacher and Holliger, 1996), *Desulfitobacterium dehalogenans* (Kruse et al., 2015) and *Sulfurospirillum multivorans* (Miller et al., 1996), OHRB belonging to the Chloroflexi (*Dehalococcoides* and *Dehalogenimonas*) do not use quinones, suggesting that different pathways are used for conserving energy via OHR (Fincker and Spormann, 2017). In the quinone-dependent electron transfer to RDases, one question remains largely unresolved, that is the generation of low redox potential electrons from the quinol/quinone redox couple (with a well-accepted E^0^’ value of -74 mV, Thauer et al., 1977). Possible mechanisms to solve this have been proposed, such as reverse electron flow in the case of *S. multivorans* (Miller et al., 1996) or electron bifurcation (Buckel and Thauer, 2013). Redox proteins involved in the electron transfer between the quinol pool and the RDases remains largely unexplored and their identification will shed light on the possible mechanisms of electron transfer in OHR.

Besides *rdhA* and *rdhB* which code for the RDase enzyme and its predicted membrane anchor, respectively, one particular gene, *rdhC*, was found in *rdh* gene clusters of several OHRB belonging to diverse phylogenetic groups. The RdhC homologs CprC, VcrC, PceC, and TmrC have been successively identified in *D. dehalogenans* (Smidt et al., 2000), *Dehalococcoides mccartyi* (Müller et al., 2004), *D. restrictus* (Maillard et al., 2005), and *Dehalobacter* sp. UNSWDHB (Jugder et al., 2016b; Wong et al., 2016), respectively. Analysis of the sequence of CprC has revealed a significant homology to proteins belonging to the NosR/NirI transcriptional regulators (Cuypers et al., 1992), suggesting that it may play a role in the regulation of the *cpr* gene cluster. Transcription analysis have shown that under OHR conditions *cprC* was transcribed with *cprD* and occasionally also as a *cprBACD* polycistronic RNA (Smidt et al., 2000), indicative for a function in the OHR metabolism. PceC is encoded in the conserved *pceABCT* gene cluster responsible for the tetrachloroethene (PCE) reductive dehalogenase activity in *D. restrictus*, but also in the genomes of *Desulfitobacterium hafniense* strain TCE1 (Maillard et al., 2005), strain Y51 (Futagami et al., 2006) and strain PCE-S (Goris et al., 2015a), as well as of less characterized OHRB (Duret et al., 2012). After the initial annotation, the role of NosR in the nitrous oxide reduction pathway was reconsidered and studies have demonstrated that it is likely playing a role in the activation of the nitrous oxide reductase (NosZ), as well as in electron transfer toward NosZ (Wunsch and Zumft, 2005; Zumft, 2005; Borrero-de Acuna et al., 2017).

PceC, and more generally RdhC proteins, are predicted to be integral membrane proteins with six transmembrane α-helices, a peripheral domain and two conserved CX_3_CP motifs. The peripheral domain harbors a conserved sequence motif for covalent binding of the flavin mononucleotide (FMN) cofactor that is also found in NosR and in two well-characterized flavoproteins, the C subunit of the Na^+^-translocating NADH-quinone reductase (NqrC) (Casutt et al., 2012; Vohl et al., 2014; Borshchevskiy et al., 2015) and in the G subunit of the *Rhodobacter* nitrogen fixation (RnfG) complex (Backiel et al., 2008; Suharti et al., 2014). Both NqrC and RnfG flavoproteins are part of large membrane-bound protein complexes involved in electron transfer and energy metabolism.

The sequence homology of PceC with NosR and other flavoproteins involved in electron transfer invited us to reconsider its function in the OHR metabolism. In this study, we present a curated selection of RdhC homologous sequences found in protein databases based on conserved sequence features. Then, the predicted membrane topology of PceC was validated with a targeted surfaceome analysis of *D. restrictus* cells. Last, we present the results of an experimental strategy developed for the heterologous production and successful reconstitution of the recombinant FMN-binding domain (rFBD) of PceC.

## Materials and Methods

### Bacterial Strains and Cultivation

*Dehalobacter restrictus* PER-K23 and *D. hafniense* TCE1 were purchased at DSMZ culture collection (Braunschweig, Germany), while *Escherichia coli* strains DH5α and BL21(λDE3) were obtained from Novagen (Merck Millipore, Schaffhausen, Switzerland). A list of main bacterial features is given in **Table 1**.

**Table 1:** Bacterial strains used in this study.

Strain	Features	Source/reference
*Dehalobacter restrictus* PER-K23	DSM 9455	Holliger et al., 1998
*Desulfitobacterium hafniense* TCE1	DSM 12704	Gerritse et al., 1999
*E. coli* DH5α	F^-^ endA1 glnV44 thi-1 recA1 relA1 gyrA96 deoR nupG purB20 φ80dlacZΔM15 Δ(lacZYA-argF) U169, hsdR17(rK^-^mK^+^), λ^-^	Novagen
*E. coli* BL21(λDE3)	F^-^ ompT gal dcm lon hsdSB (Rb^-^mB^-^) λ(DE3 [santi lacUV5-T7 gene 1 ind1 sam7 nin5])	Novagen

*Dehalobacter restrictus* PER-K23 and *D. hafniense* TCE1 were cultivated anaerobically with hydrogen and tetrachloroethene (PCE) as electron donor and acceptor, respectively, as previously described (Prat et al., 2011; Comensoli et al., 2017).

*Escherichia coli* strains were routinely cultivated in Luria-Bertani liquid and solid media. For large-scale production of rFBD protein, *E. coli* BL21(λDE3) harboring the pFBD plasmid was cultivated in ZYM-5052 medium following the protocol established by Studier (2014). Antibiotics were supplemented depending on the plasmid, as indicated in **Table 2**.

**Table 2:** Plasmids used in this study.

Plasmid	Features	Source/reference
pET24-d	IPTG-inducible T7 promoter, kanamycine^R^ (50 μg/mL), C-terminal His_6_-tag	Novagen
pFBD	pET24d expressing FMN-binding domain of PceC (aa 41–200)	This study
pFBD-T168V	pFBD with single mutation expressing a valine variant of Thr168	This study
pETDuet-1	IPTG-inducible T7 promoter, ampicilline^R^, two multiple cloning sites, MCS1 with C-terminal His_6_-tag, MCS2 with C-terminal S∙tag^TM^	Novagen
pFTP1	pETDuet-1 expressing Ftp1 from MCS2	This study
pFTP2	pETDuet-1 expressing Ftp2 from MCS2	This study

### Plasmid Construction

#### Polymerase Chain Reaction (PCR)

Standard PCR reaction mixtures for cloning consisted of a 50-μL reaction containing 5 μL Pfu DNA polymerase 10× buffer (Promega, Dübendorf, Switzerland), 75 μM dNTPs, 0.5 μM each primer and 0.5 μL of Pfu DNA polymerase. The DNA was amplified in T3 Biometra thermocycler (LabGene, Châtel-Saint-Denis, Switzerland) with the following steps: 2 min initial denaturation at 95°C, 30 cycles of 1 min denaturation at 95°C, 1 min of primer annealing at 52°C, 1 min of elongation at 72°C, and 10 min of final extension at 72°C.

Polymerase chain reaction reactions for site-directed mutagenesis consisted of 5 μL Pfu Turbo polymerase 10× buffer (Agilent Technologies, Morges, Switzerland), 12.5 μM each primer, 75 μM dNTPs, 50 ng of plasmid, 1 μL Pfu Turbo polymerase. The program used was the following: 30 s initial denaturation at 95°C, 30 cycles of 30 s denaturation at 95°C, 60 s of primer annealing at 55°C, 10 min of elongation at 68°C, and a final 7 min extension step at 68°C.

Polymerase chain reaction products for cloning were cleaned using the QIAquick PCR Purification kit (Qiagen AG, Hombrechtikon, Switzerland), following manufacturer’s instructions. PCR products and other DNA samples were quantified with the NanoDrop 1000 apparatus (Life Technologies Europe B.V., Zug, Switzerland).

#### Cloning

A list of the plasmids and oligonucleotides used in this study is given in **Tables 2, 3**, respectively.

**Table 3:** Oligonucleotides used in this study.

Primer name	5′–3′ sequence	Features
FBD-24-F	GCGCCCATGGGACAATCGGTTGATTACAAGGGAATC	*Nco*I site
FBD-24-R	GCGCCTCGAGTAAATCGTAAGGGTTGGCCCATTG	*Xho*I site
FBD-T168V-F	ACGGTAACAGGTTCAGTAGTGTCGTCACATGCT	Thr-Val
FBD-T168V-R	AGCATGTGACGACACTACTGAACCTGTTACCGT	Thr-Val
pET24-F2	GGTGATGTCGGCGATATAGG	Sequencing
pET24-R2	CGTTTAGAGGCCCCAAGG	Sequencing
FTP1-F	GCGCCATATGAATGGGAAACCTGTACAACAG	*Nde*I
FTP1-R	GCGCCTCGAGATCTTTGACGAATTCGTACTC	*Xho*I
FTP2-F	GCGCCATATGTTGTCTGCAGAGACCAAGG	*Nde*I
FTP2-R	GCGCCTCGAGTTTGCTTTCTGGGGAAGGTGTC	*Xho*I
Duet-MCS2-F	TTGTACACGGCCGCATAATC	Sequencing
Duet-MCS2-R	GCTAGTTATTGCTCAGCGG	Sequencing

The coding sequence for the FMN-binding domain (FBD) of PceC was amplified by PCR using genomic DNA from *D. hafniense* TCE1 and the primers FBD-24-F/R. The PCR product and the vector pET24d were digested with *Nco*I and *Xho*I in a 40-μL reaction mixture containing 4 μL of buffer D, 0.4 μL of BSA, 1 μL of each enzyme (all components from Promega) which was incubated at 37°C for 2 h. The digested vector was then incubated 15 min after addition of 1 μL of thermosensitive alkaline phosphatase (Promega). Both digested PCR product and vector were purified with the QIAquick PCR Purification kit (Qiagen) and eluted in 30 μL of ddH_2_O. After DNA quantification, the ligation reaction was set-up as follows: a 10-μL reaction consisted of 1 μL of T4 DNA ligase (Roche, Sigma-Aldrich, Buchs, Switzerland), 1 μL of 10 mM ATP, insert and vector DNA in 3:1 molar ratio and 1 μL of T4 DNA ligase (Roche). The reaction was incubated for 2 h at room temperature. Five μL of the ligation reaction was directly transformed by heat-shock into 50 μL of RbCl-competent *E. coli* DH5α cells, following standard protocol (Sambrook et al., 1989). Positive transformants were selected by colony PCR using primers pET24d-F2/R2. Plasmids were recovered from overnight 10-mL *E. coli* cultures using QIAprep Spin Miniprep kit (Qiagen) and sequenced for verification as described previously (Duret et al., 2012). The resulting plasmid was named pFBD and used for the production of the C-terminal His_6_-tagged rFBD protein.

Cloning of the coding sequence for Ftp1 and Ftp2 was done with the same procedure by using primers FTP1-F/R and FTP2-F/R, respectively, the vector pETDuet-1 and the restriction enzymes *Nde*I and *Xho*I. Sequence verification of the plasmids was done by colony PCR with the primers Duet-MCS2-F/R. The resulting plasmids were named pFTP1 and pFTP2, respectively and used for the production of rFtp1 and rFtp2 proteins with C-terminal S∙tag^TM^.

#### Site-Directed Mutagenesis

The T168V variant of rFBD was obtained by applying the QuikChange site-directed mutagenesis protocol established by Stratagene with the plasmid pFBD and the primers FBD-T168V-F/R. The resulting PCR product was digested for 1 h at 37°C with 1 μL of *Dpn*I (Promega), prior to transformation into *E. coli* DH5α as described above. The mutation was verified by sequencing.

### Sequence Analysis

The following software were used to analyze and compare the sequences of RdhC proteins: ClustalX2.0 for sequence alignment (Larkin et al., 2007); iTOL for drawing sequence likelihood trees (Letunic and Bork, 2016); Weblogo for sequence motifs (Crooks et al., 2004); CCTOP for topology prediction (Dobson et al., 2015) and TOPO2 for graphic representation of the topology (developed by S. J. Johns^1^).

The procedure for the identification and selection of RdhC homologous sequences from protein databases is presented in section “Sequence Analysis of RdhC Proteins” of the Supplementary Material.

### Surfaceome Analysis

#### Biomass Collection and Sample Preparation

For surfaceome analysis, the biomass from a 200-mL culture of *D. restrictus* PER-K23 was harvested by centrifugation when approximately 70% of PCE was consumed. The biomass was collected by 30 min centrifugation at 1000 × *g* and 4°C. After carefully decanting the supernatant, the biomass pellet was resuspended in 10 mL ice-cold wash buffer (20 mM Tris-HCl, pH 7.5, 150 mM NaCl) by stirring gently the tube to avoid cell lysis. The biomass was collected by 15 min centrifugation as before and washed twice more. The pellet was then resuspended in 4 mL of digestion buffer (wash buffer supplemented with 0.1 M arabinose and 10 mM CaCl_2_). Prior to adding the trypsin (10 μL of trypsin (Gold-Mass spec grade, Promega at 1 mg/mL in digestion buffer), the biomass suspension was split into two samples in 15-mL Falcon tubes. The trypsin was added to one of the samples (‘shaved’ sample), while the other one served as negative control. Both tubes were incubated for 15 min at 37°C by stirring at 120 rpm, then placed on ice for 5 min and centrifuged for 10 min at 1000 × *g* and 4°C. The supernatant was collected, filtered at 0.22 μm and flash-frozen in liquid nitrogen. In order to produce a membrane reference sample, the pellet from the control sample was resuspended in 2 mL of wash buffer and the biomass was lyzed by sonication with 10 cycles of 10× 1 s pulses at 60% amplitude on Sonic Dismembrator FB120 (Fisher Scientific, Reinach, Switzerland). After 5 min centrifugation as above, unbroken cells were discarded and the membranes were obtained from the supernatant by ultracentrifugation for 20 min at 100,000 × *g* and 4°C. The resulting pellet (membrane fraction) was resuspended in 2 mL of wash buffer, flash-frozen in liquid nitrogen. All samples were stored at -80°C until further analysis.

#### In-Solution Digestion

‘Shaved’ and ‘control’ samples were reconstituted in 4 M Urea, 10% acetonitrile and buffered with Tris-HCl pH 8.5 to a final concentration of 30 mM. Proteins were reduced, alkylated, and digested using trypsin as previously described (Dalla Vecchia et al., 2014). Total membrane lysate was heated 10 min at 80°C in Rapigest SF surfactant 0.2% and sonicated 5 min in order to increase the solubility of hydrophobic proteins. Proteins were buffered with Tris-HCl pH 8.5 to a final concentration of 30 mM and reduced using 10 mM dithioerythritol (DTE) at 37°C for 60 min. Proteins were incubated in 40 mM iodoacetamide at 37°C for 45 min in the dark and the reaction was further quenched by the addition of DTE to a final concentration of 10 mM. Protein lysate was first diluted threefold using ammonium bicarbonate at 50 mM and samples were digested overnight at 37°C using 1 μg of mass spectrometry grade trypsin gold and 10 mM CaCl_2_. Rapigest was cleaved by the addition of trifluoroacetic acid 10% (final pH < 2) and incubated 45 min at 37°C. Peptides from the shaving experiment and the total membrane lysate were desalted in StageTips using 6 disks from an Empore C18 (3 M) filter based on the standard protocol (Rappsilber et al., 2007). Purified samples were dried down by vacuum centrifugation and stored at -20°C.

#### Mass Spectrometry and Data Analysis

Samples were resuspended in 2% acetonitrile containing 0.1% formic acid for LC–MS/MS injections. Reverse phase separation were performed on a Dionex Ultimate 3000 RSLC nano UPLC system connected online with an Orbitrap Lumos Fusion Mass-Spectrometer. To ensure a robust detection of Dehre_2396 peptides (PceC), an inclusion list corresponding to the expected peptides was established using Skyline 3.1.0.7312 and was further included in the data acquisition method. Raw data was processed using MS-Amanda (Dorfer et al., 2014) and SEQUEST in Proteome Discoverer v.1.4 against the proteome of *D. restrictus* PER-K23. Spectra were searched with a fragment ion mass tolerance of 0.050 Da and a parent ion tolerance of 10.0 PPM. Carbamidomethylation of cysteine residues was specified as a fixed modification. Glutamine to pyro-glutamate of N-termini, oxidation of methionine residues, phosphorylation of serine, threonine, and tyrosine residues and FMN covalently bound to threonine residues were specified as variable modifications. Data was further processed by X!tandem, inspected in Scaffold 4 and spectra of interest were manually validated.

### Heterologous Protein Production and Purification

#### Production and Purification of Recombinant FMN-Binding Domain of PceC (rFBD)

An overnight pre-culture (37°C and 180 rpm) of *E. coli* BL21(λDE3) harboring the pFBD plasmid was done in 50 mL LB medium. Starting from here, two different batches of rFBD production were performed as follows. For the first batch (P1), 2 L of LB medium were inoculated with 20 mL of pre-culture (1:100 dilution) and cultivated until the optical density (OD_600__nm_) reached approximately 1.0. IPTG was added at 0.1 mM (final concentration) to induce the protein production and the culture was further incubated for 2 h in the same conditions. OD_600__nm_ reached a value of 2.5 and 6 g of (wet weight) biomass was collected. For the second batch (P2), 2 L of ZYM-5052 (auto-induction) medium were inoculated with 2 mL of pre-culture (1:1000 dilution) and cultivated for 16 h at 20°C and 250 rpm. Cell density reached a value of 10 and 20 g of biomass was collected.

Purification of rFBD inclusion bodies from the collected biomass was applied as follows. The biomass pellet was resuspended in lysis buffer [50 mM Tris-HCl, pH 7.5, 100 mM NaCl, SigmaFast protease inhibitors (Sigma-Aldrich), a few DNase crystals (Roche)] at 10 mL per g of biomass. After three cycles of French press (1000 PSI), the lysate was centrifuged 5 min at 500 × *g* ant 4°C and unbroken cells were removed. The supernatant was centrifuged 15 min at 12,000 × *g* and 4°C. The resulting pellet was rinsed in wash buffer [50 mM Tris-HCl, pH 7.5, 100 mM NaCl, 1 mM EDTA, 1% (w/v) Triton X-100 and 1 M urea], and inclusion bodies were resuspended in solubilization buffer (50 mM Tris-HCl, pH 7.5, 100 mM NaCl, supplemented with 4–8 M urea, depending on the experiments) at 10 mL/g. After one freeze-thaw cycle at -20°C, the suspension was centrifuged at 12,000 × *g* as above and the supernatant containing solubilized rFBD protein was collected.

#### Production of Recombinant Flavin-Trafficking Proteins (rFtp)

Recombinant rFtp1 and rFtp2 proteins were produced in *E. coli* BL21(λDE3) similarly as rFBD with the following changes. Starting from an overnight pre-culture, 1 L of each culture was performed as above, but induction was achieved by 3 h incubation at 30°C. Each culture reached an OD_600__nm_ value of 2.9, corresponding to 3.4 and 3.6 g of biomass, respectively. After cell lysis and fractionation as presented above, soluble cell-free extracts were obtained and used for reconstitution experiments.

Protein concentration was estimated with the Pierce BCA assay (Thermo Fisher Scientific, Lausanne, Switzerland) following the manufacturer’s instructions. Calibration curve was done with BSA in the same buffer conditions as the analyzed samples.

### Reconstitution of rFBD Proteins

#### Reconstitution by Reverse Urea Gradient

One sample of urea-denatured rFBD protein (obtained from 1 g of biomass sample P1, see above) was loaded on a 1-mL His-Trap Ni-NTA affinity column connected to ÄKTAprime plus^TM^ system (GE Healthcare, Glattbrugg, Switzerland) in buffer A (50 mM Tris-HCl, pH 7.5, 150 mM NaCl, 25 mM imidazole, 1 mM dithiothreitol) supplemented with 4 M urea (buffer A+). After extensive removal of unbound proteins with buffer A+, the column was disconnected from the system and a 10-step reverse urea gradient was manually applied by injecting one column volume (CV) of buffers with decreasing concentration of urea (see Supplementary Table 1 for details). Then, the column was further rinsed with 15 CV of buffer A before rFBD protein was eluted by injection of 10 successive CV of buffer B (buffer A supplemented with 0.6 M imidazole). Ten μL aliquots were run on SDS-PAGE following standard protocol (Sambrook et al., 1989), then FMN-containing proteins were detected under UV illumination. Last, the gel was stained with Coomassie Blue [0.1% (w/v) Coomassie Blue R250 in 10% (v/v) acetic acid and 40% (v/v) ethanol].

#### Reconstitution by Stepwise Dialysis

Inclusion bodies were purified from 5 g of *E. coli* producing rFBD (biomass sample P2, see above) and resuspended in solubilization buffer containing 8 M urea. After 15 min centrifugation at 4500 × *g* and 4°C, the solubilized and urea-denatured rFBD sample (20 mL) was filtered at 0.45 μm and transferred to a dialysis tube (Spectra/Por with 6-8000 MWCO, Spectrum Labs, Breda, Netherlands) in 2 L dialysis buffer (50 mM Tris-HCl, pH 7.5, 150 mM NaCl, 1 mM dithiothreitol) supplemented with 4 M urea. Dialysis was performed for 2 h at room temperature. The sample was collected from the tube, supplemented with 1.4 mg of rFtp1-containing protein extract, 5 mM MgSO_4_ and 1 mM FAD, incubated for 20 min at room temperature, and transferred to a fresh dialysis tube. Dialysis was done as before in a buffer containing 2 M urea. The sample was collected, supplemented with rFtp1, then incubated for 20 min and dialysed as before in a buffer lacking urea. Last, the sample was dialysed overnight in fresh buffer to remove residual urea and excess of FAD. A volume of 25 mL of soluble rFBD protein was recovered in the supernatant after 20 min of centrifugation at 15,500 × *g* and 4°C and its concentration estimated at 1.9 mg/mL. Aliquots of each step were analyzed by SDS-PAGE as described above.

### Mass Spectrometry Analyses of rFBD Proteins

The methods used for mass spectrometry analysis of rFBD protein are presented in Section “Mass Spectrometry Analyses of the Reconstituted rFBD Protein” of the Supplementary Material.

## Results

### Sequence Analysis of PceC

**FIGURE 1:**
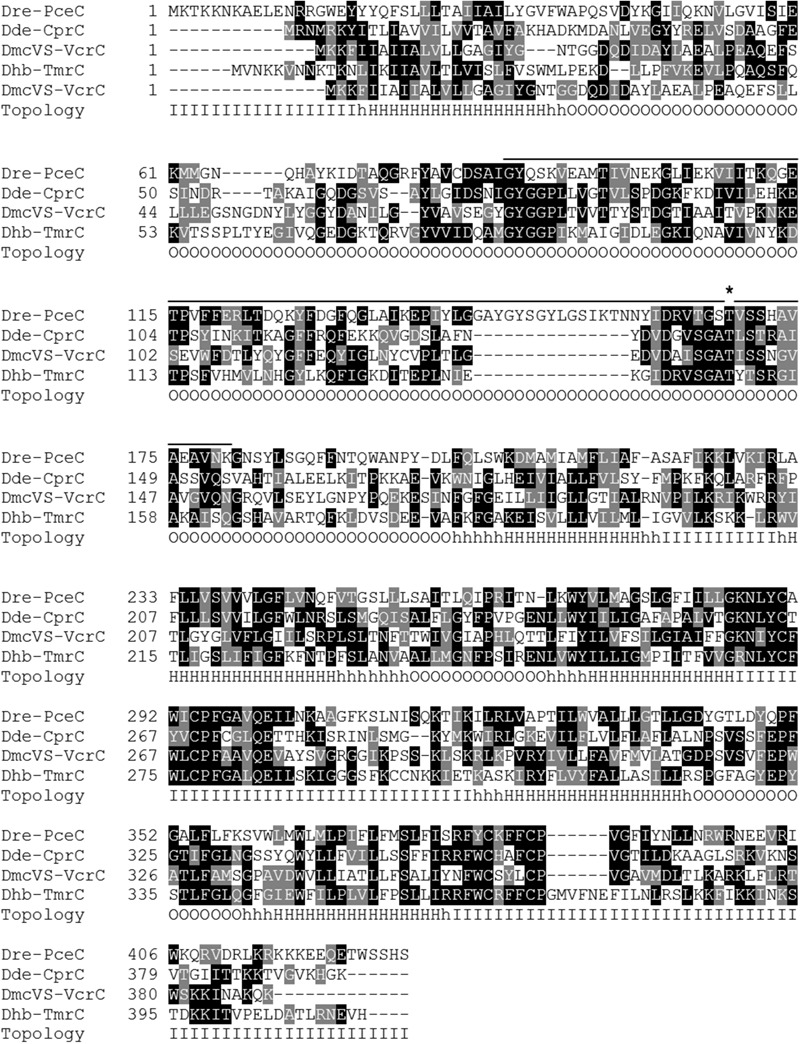
Sequence alignment of four typical members of the RdhC enzyme family: PceC (CAG70347.1) of *Dehalobacter restrictus* (Dre); CprC (AAD44543.2) of *Desulfitobacterium dehalogenans* (Dde); VcrC (ACZ62389.1) of *Dehalococcoides mccartyi* strain VS (DmcVS); TmrC (WP_021315090.1) of *Dehalobacter* sp. strain UNSWDHB (Dhb). The predicted topology is indicated under the alignment: O, outside; I, inside; H, transmembrane α-helices. The bold line above the alignment indicates the predicted FMN-binding domain (FBD, smart00900) with the conserved threonine residue predicted to bind FMN covalently (indicated by the asterisk).

The alignment of the four reported RdhC sequences reveals important conserved features (**Figure 1**). Topology and conserved domain predictions suggest the presence of six transmembrane α-helices and a peripheral FBD (FMN-bind, smart00900) located on the outside of the cytoplasmic membrane between helix 1 and 2. The FBD of RdhC sequences shows similarity to NqrC, a subunit of the NADH:quinone oxidoreductase. NqrC has been reported to bind FMN covalently (Nakayama et al., 2000; Borshchevskiy et al., 2015).

Additional sequence homology and protein domain architecture analysis revealed some similarity between PceC and the functionally characterized membrane-bound proteins NosR and NapH (Supplementary Figure 1). PceC displays most features of NosR (Wunsch and Zumft, 2005) but harbors a shorter extra-cytoplasmic loop and lacks Fe–S clusters at the C-terminal end. The C-terminal end of PceC also shows similarities to the domain architecture of the nitrate reductase membrane-bound subunit NapH, which together with NapG is playing a role in transferring electrons from menaquinones to NapA (Kern and Simon, 2008).

### Diversity of RdhC Sequences and Definition of the RdhC Protein Family

**FIGURE 2:**
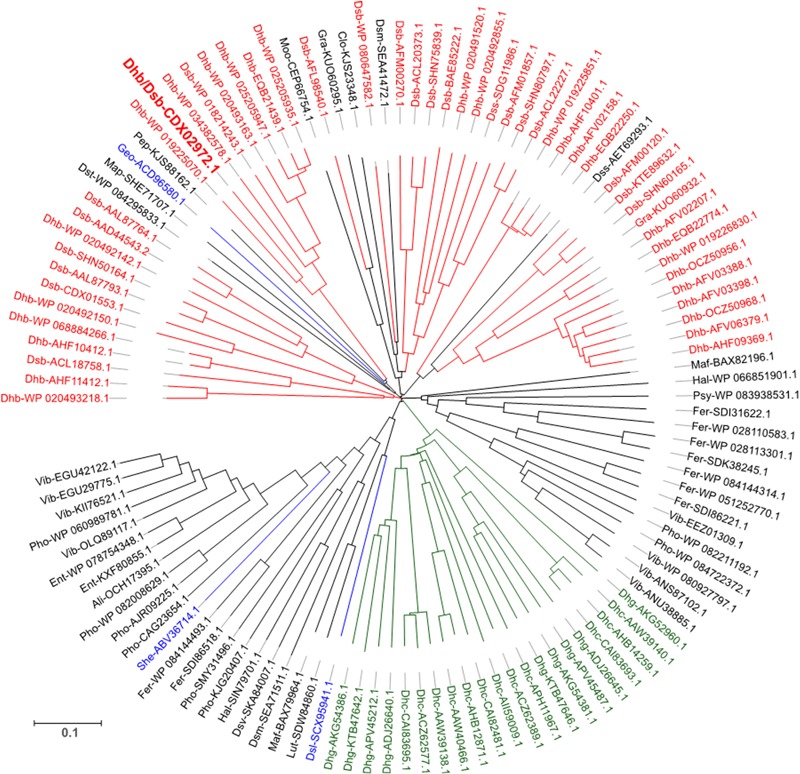
Sequence likelihood analysis of 117 representative RdhC proteins. Sequences are annotated with an abbreviation of the corresponding species followed by the reference number. The sequence annotated in bold red is PceC from *Desulfitobacterium hafniense*, the representative sequence for the protein under investigation in this study. Colors indicate sequences belonging to well-described OHR bacterial genera: red, *Dehalobacter* and *Desulfitobacterium*; green, *Dehalococcoides* and *Dehalogenimonas*; blue, additional OHRB. Detailed information on the overall database used is available in Supplementary Material (Supplementary Table 2).

Looking at the diversity of RdhC sequences in databases, a first selection of 433 sequences was obtained by sequence homology analysis (see section “Sequence Analysis of RdhC Proteins” of the Supplementary Material for a detail description of the selection procedure). Sequence alignment and clustering with 95% identity has reduced the number to 236 unique clusters. From this selection, only those coming from genomes harboring at least one *rdhA* gene were considered. This new selection delivered 117 RdhC clusters comprising a total of 199 unique sequences, each cluster displaying between 1 and 9 unique sequences (see Supplementary Table 2). Sequence likelihood analysis of RdhC clusters is displayed in **Figure 2**. A total of 71 RdhC clusters covering 135 unique sequences come from known OHRB. Among them, with the exception of PceC which is found in both *Dehalobacter* and *Desulfitobacterium*, each RdhC cluster is exclusively found in one specific genus. *Dehalobacter* displays the highest number of clusters (28 clusters with 42 unique sequences), while 20 clusters (30 sequences) are found in *Desulfitobacterium*. *Dehalococcoides*, and *Dehalogenimonas*, the *Chloroflexi* members of OHRB, display 12 and 9 clusters, respectively, comprising 45 and 10 unique sequences (Supplementary Table 2). Three additional RdhC clusters were found in newly identified OHRB: *Geobacter lovleyi, Shewanella sediminis*, and *Desulfoluna spongiiphila*. The remaining 43 clusters (88 unique sequences) belong to bacteria that have not been recognized as OHRB yet, suggesting that the reservoir for new OHRB remains largely unexplored. Among them, three bacterial genera harbor 25 clusters with 37 unique sequences (16 sequences for the genus *Vibrio*, 11 for *Photobacterium*, and 10 for *Ferrimonas*) making them interesting candidates to expend the phylogeny of OHRB. All three genera belong to marine Gammaproteobacteria, suggesting that, while OHRB were mostly isolated from sediments of contaminated sites, marine environments represent an important ecological niche for OHRB.

**FIGURE 3:**
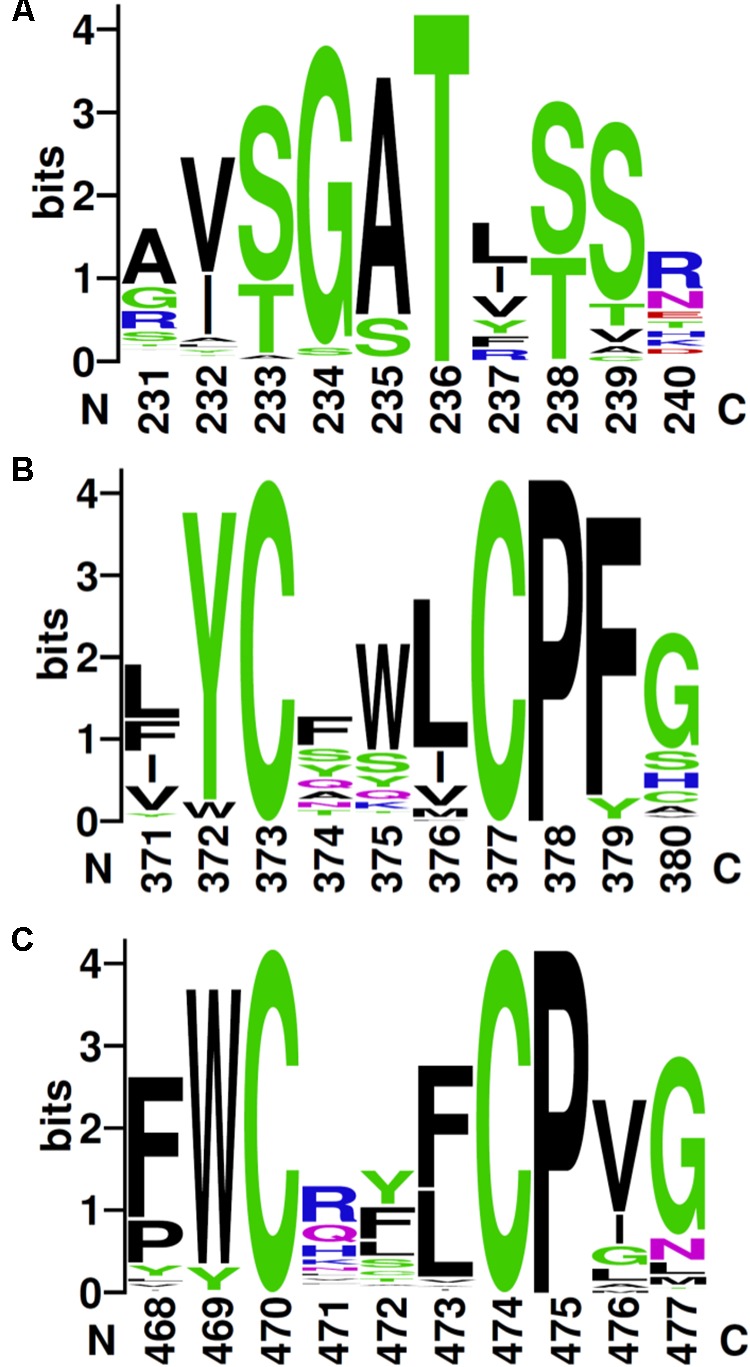
Conserved sequence motifs in RdhC family. Based on the alignment of the 117 RdhC non-redundant representative sequences, motifs were drawn using Weblogo: **(A)** the FMN-binding motif, with the fully conserved threonine involved in binding FMN covalently; **(B,C)** the two CX_3_P signatures. Residue numbering follows the alignment.

The alignment of the 117 unique sequences revealed three well-conserved sequence motifs, which are defining the RdhC family (**Figure 3**). In the FMN-binding motif (**Figure 3A**), the hydroxyl side chain of the fully conserved threonine is predicted to covalently bind the cofactor in a phosphoester-threonyl-FMN bond (Backiel et al., 2008). A consensus for the FMN-binding motif in RdhC sequences is proposed here as [S/T]G[A/S]TX[S/T], similarly to the motif proposed earlier (Deka et al., 2015). The two other conserved motifs are of the type CX_3_CP, for which, however, no function has been yet assigned (**Figures 3B,C**).

### Experimental Validation of PceC Topology and FMN-Binding

**FIGURE 4:**
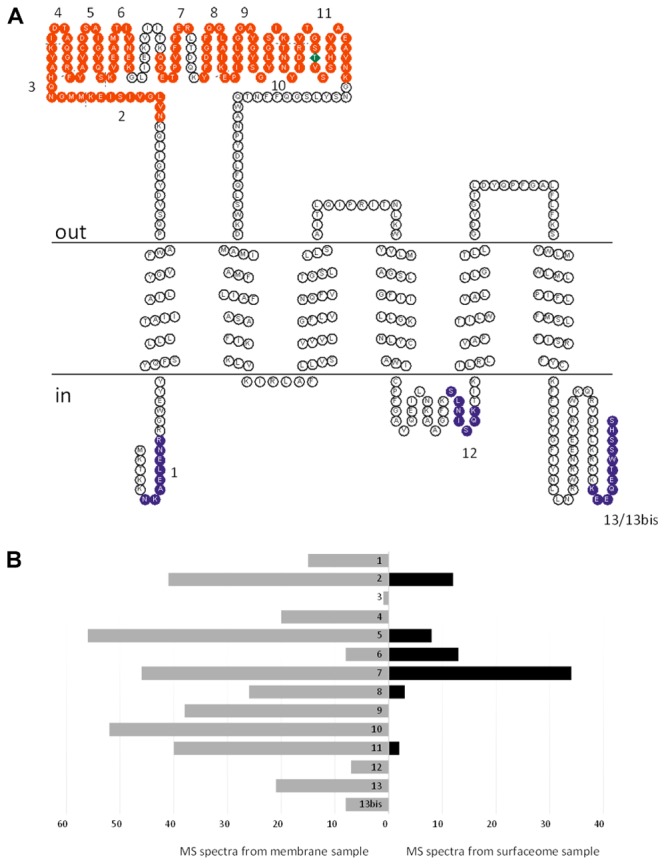
Topology analysis of *D. restrictus* PceC from shaving experiment. **(A)** The predicted topology of PceC is shown. In red are indicated the peptides detected at the surface of *D. restrictus* cells, while in purple the ones exclusively detected in the membrane sample. **(B)** Graphical view of the number of detected peptides in the membrane sample (in gray) and in the surfaceome (in black).

The FBD of PceC was predicted to face the outside of the cytoplasmic membrane. In order to validate the prediction, a peptide shaving experiment was done with whole cells of *D. restrictus* (**Figure 4**). Proteomic analysis of the cell surface (so-called surfaceome) clearly showed that six peptides of the FBD were detected, while none of the peptides predicted to be located in the cytoplasmic loops were identified. In contrast, three of the cytoplasmic-oriented peptides were detected in the control membrane sample. This unambiguously demonstrates that the FMN-binding peripheral domain of PceC is exposed to the exocytoplasmic side of the membrane.

**FIGURE 5:**
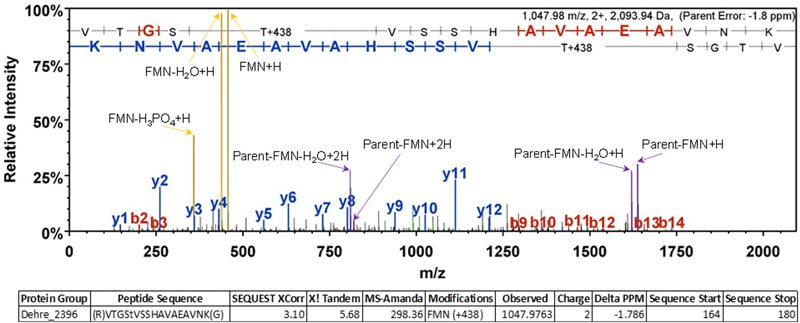
Representative MS/MS spectrum confirming the FMN localization at position T168 of PceC. Diagnostic ions corresponding to the FMN+H and its fragments are highlighted in yellow allowing unambiguous identification of the post-translational modification.

The presence of a covalently-bound FMN in PceC was investigated using a crude membrane fraction from a culture of *D. restrictus* PER-K23. In-solution digested proteins were further analyzed by LC–MS/MS. Multiple detections of the peptide containing the expected modified threonine were achieved (**Figure 5**). The high quality of the spectra allowed a clear detection of FMN+H and its expected fragments as described previously (Guyon et al., 2008). All these evidences confirmed the presence of the FMN at position T168 of PceC.

### Heterologous Production of the FMN-Binding Domain of PceC (rFBD)

The highly hydrophobic nature of PceC prevents its production in a soluble form. Therefore, it was decided to produce the peripheral FBD of PceC in *E. coli*. From sequence prediction analysis, the FBD of PceC from *D. hafniense* TCE1 was defined as the region between residues 41 and 200 (Supplementary Figure 2). The corresponding DNA sequence was cloned in fusion with a C-terminal His_6_-tag giving the plasmid pFBD (see **Table 2**). Early attempts revealed that the rFBD was prone to strong protein aggregation in *E. coli* (Supplementary Figure 3A), suggesting that denaturation and refolding are necessary to produce rFBD in a soluble form. Addition of flavins during protein production did not produce any soluble rFBD either (Supplementary Figure 3B). Using a strategy of auto-induction (Studier, 2014), large amount of rFBD was produced in form of inclusion bodies in *E. coli* (Supplementary Figure 3C). Inclusion bodies were easily recovered by fractionation of the lysed *E. coli* biomass and efficiently solubilized in buffer containing 4–8 M urea. Refolding attempts using various strategies such as reverse urea gradient using affinity chromatography or dialysis with decreasing concentration of urea were unsuccessful (data not shown). Recent literature on flavoproteins which display covalently-bound FMN (Bertsova et al., 2013; Deka et al., 2016; Zhang et al., 2017) have suggested that these proteins required a helper protein for efficient assembly of the FMN cofactor and successful folding.

### Identification and Production of Flavin-Trafficking Proteins (Ftp) From *D. hafniense* TCE1

Flavin-trafficking proteins [Ftp, previously named ApbE (Deka et al., 2015)] are FAD-binding proteins, from which two classes have been defined. One of them shows FAD hydrolysing activity and is able to deliver FMN to flavoproteins (Deka et al., 2016). Two Ftp encoding genes were identified in the genome of *D. hafniense* TCE1 with locus number DeshaDRAFT_4346 and _4351^2^. Both genes are located in a multi-gene cluster with no clear function. One gene of this cluster is coding for a predicted FMN-binding lipoprotein (DeshaDRAFT_4350), suggesting that the Ftp proteins are primarily involved in the maturation of this protein (Supplementary Figure 4A). Sequence analysis of both Ftp proteins revealed the presence of a clear lipoprotein signal peptide similarly to Ftp of *Treponema pallidum* which was proposed to be anchored in the cytoplasmic membrane facing the periplasmic side of the cell (Deka et al., 2013). Conserved residues suggest that both Ftp of *D. hafniense* belong to the Mg^2+^-dependent hydrolysing class (Supplementary Figure 4B) and are thus likely to be involved in the maturation of flavoproteins.

Initially, both *ftp* genes of *D. hafniense* TCE1 were cloned for heterologous expression in *E. coli*. In order to avoid protein aggregation, the coding sequence for the predicted lipid anchor of Ftp was omitted, as shown for Ftp1 (Supplementary Figure 5). Both recombinant Ftp (rFtp1, rFtp2) were successfully produced in a soluble form (Supplementary Figure 6). Soluble protein extracts of *E. coli* cells producing rFtp1 was further used to reconstitute rFBD protein.

### Reconstitution of rFBD Proteins

The rFBD protein was obtained from purified inclusion bodies, denatured with urea as presented above, and subjected to different reconstitution strategies with the help of rFtp1. Initially, a simple dilution experiment was performed with both rFtp1 and rFtp2 extracts (see detail description of the method in Section “Reconstitution by Dilution” of the Supplementary Material). UV illuminated gels showed a fluorescent signal at the expected size of rFBD, however very weak (data not shown). Therefore, two other reconstitution strategies were applied using rFtp1 where care was taken to remove urea more extensively.

#### Reconstitution by Reverse Urea Gradient on Ni-NTA Column

**FIGURE 6:**
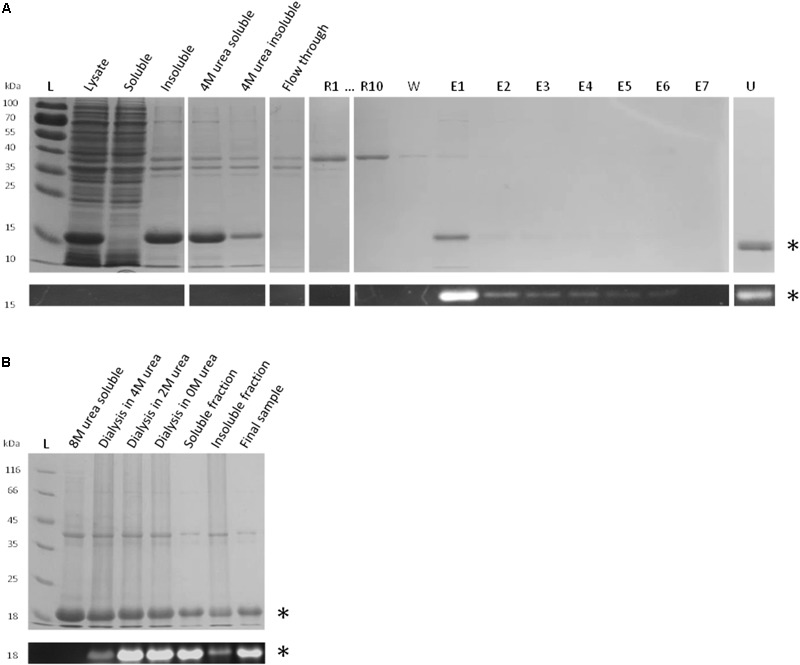
Reconstitution strategies for rFBD. **(A)** Reconstitution of rFBD by reverse urea gradient on Ni-NTA column. For the reconstitution steps, only the first (R1) and the last sample (R10) are shown. Legend: L, protein ladder; R#, 10 successive gradient steps with decreasing urea concentration; W, wash step; E#, successive eluted samples; U, sample eluted with 4 M urea. **(B)** Reconstitution of rFBD by dialyzing out urea in a stepwise manner. For both **(A,B)**, the top panels show Coomassie blue stained gels, while the bottom panels display the same gels under UV illumination. Stars indicate rFBD proteins.

Urea-denatured rFBD protein (corresponding to 1 g of P1 biomass) was reconstituted on column by gradually removing urea in presence of FAD and rFtp1 cell extract. Samples collected during this experiment were analyzed by SDS-PAGE, Coomassie blue staining and UV illumination (**Figure 6A**). While most of the denatured rFBD protein was bound to the column, the elution pattern after reconstitution suggested that only a relatively small portion of it could be recovered (mostly in sample E1 with 0.22 mg protein). However, the protein in this sample was fully soluble (data not shown) and emitted a strong fluorescent signal, indicative for a successful assembly of the FMN cofactor. Some residual protein remained attached to the column (sample U in **Figure 6A**), which also prevented the recovery of significant amount of soluble and reconstituted rFBD protein.

#### Reconstitution by Stepwise Dialysis

In order to improve the yield and scale up the reconstitution procedure, a strategy with stepwise dialysis was developed. Inclusion bodies from 5 g of P2 biomass were solubilized in 8 M urea and subjected to successive dialysis with decreasing concentrations of urea. FAD and rFtp1 were also added in the dialysis tube at each step. The level of fluorescence observed along the successive dialysis steps shows that flavin transfer occurs already in presence of 4 M of urea, suggesting a significant robustness of rFtp1. The fluorescent signal further increased with decreasing urea concentrations (**Figure 6B**). While a significant amount of protein remained insoluble, approximately 50 mg of reconstituted soluble rFBD protein could be produced with this strategy.

### Characterization of the Reconstituted rFBD Protein

#### Reconstituted rFBD Protein Displays One Covalently-Bound FMN Cofactor

Mass spectrometry analysis of the mass of rFBD gave further evidence for the presence of covalently-bound FMN. A clearly dominating mass of 19,212.4 Da was detected for rFBD, which corresponds to the theoretical mass of rFBD (18,905.3 Da), from which the initial methionine was cleaved off (18,774.1 Da), and which contains one FMN (456.3 Da) attached to the threonine residue (H_2_O was released upon flavinylation, -18 Da) (Supplementary Figure 7). This results in a theoretical mass of 19,212.4 Da, fully matching with the observed mass. It is also worth noting that, although the analysis was not quantitative, no mass corresponding to non-flavinylated rFBD protein was detected, suggesting that the yield of flavinylation was nearly 100% in rFBD present in the soluble fraction.

#### FMN Is Covalently Bound to the Predicted Threonine in rFBD

As suggested by sequence alignment and prediction (**Figure 3A**), threonine-168 of PceC is likely to be involved in binding FMN covalently. The reconstituted rFBD protein was subjected to top–down mass spectrometry analysis which could localize FMN on the string of four residues (GSTV) of rFBD (Supplementary Figure 8 and Supplementary Tables 3, 4), where the threonine (residue 129 in rFBD) corresponds to threonine-168 of the full-length PceC sequence.

**FIGURE 7:**
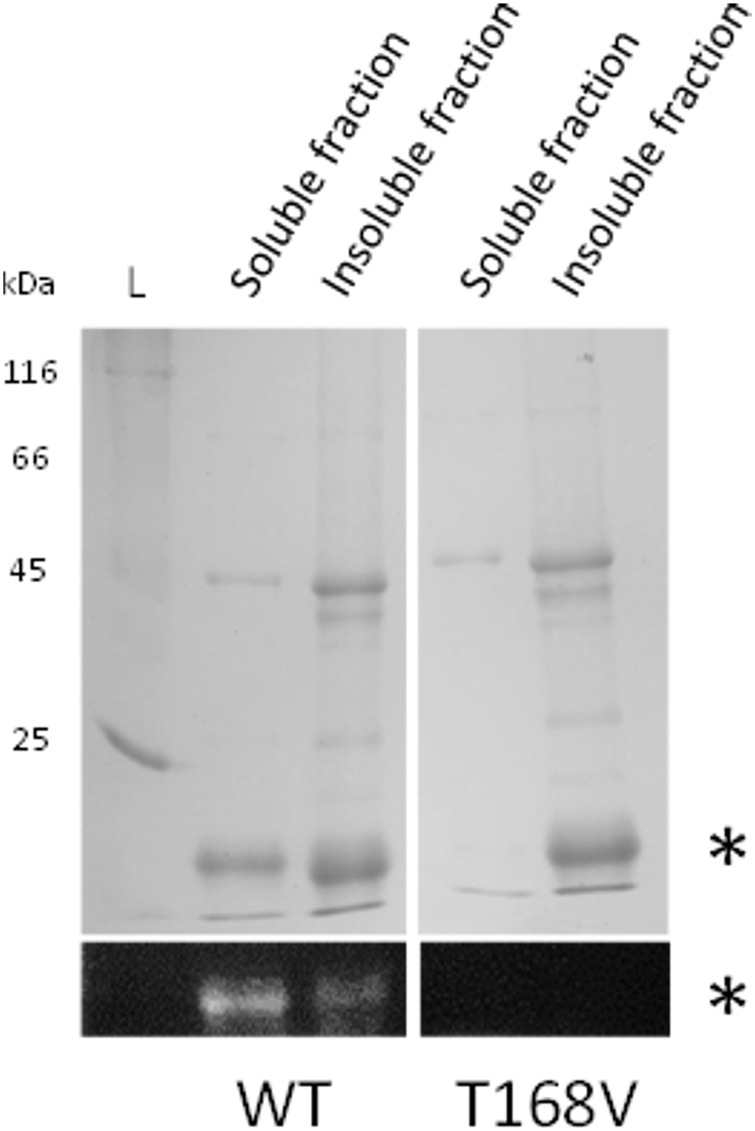
Reconstitution of rFBD wild-type (WT) and threonine-168 variant (T168V). The top panels show Coomassie blue stained gels, while the bottom panels display the same gels under UV illumination. Stars indicate rFBD proteins. L, protein ladder.

Additional evidence for this was obtained after reconstitution was applied to a valine variant of the conserved threonine (**Figure 7**). In contrast to rFBD wild-type protein, the valine variant could not be loaded with FMN and was only found in the insoluble fraction after reconstitution. This unambiguously confirmed that threonine-168 of PceC (threonine-129 of rFBD) is involved in FMN-binding, and further highlights that FMN insertion is required for refolding rFBD protein into a soluble protein.

## Discussion

Among the variety of genes found in *rdh* gene clusters of OHRB (Kruse et al., 2016), the product of only few genes (RdhA, RdhK, and RdhT) have been functionally characterized. PceC, and more generally RdhC proteins are encoded in many *rdh* gene clusters identified in organohalide-respiring Firmicutes. Typically, genomes of *Desulfitobacterium* spp. harbor an *rdhC* homolog in most of *rdh* gene clusters (Kruse et al., 2017), while it is present in 10 out of 24 clusters in *D. restrictus* (Kruse et al., 2013; Rupakula et al., 2013). Our conservative survey for RdhC sequences present in protein databases highlights the presence of *rdhC* in *rdh* gene clusters belonging to other phyla than the Firmicutes, such as the Chloroflexi, the Proteobacteria, and the Bacteriodetes. This strongly suggests that, although RdhC appears to be dispensable for OHR metabolism in many bacteria, it must have emerged early in the evolution of *rdh* gene clusters.

Sequence analysis of RdhC and their corresponding RdhA in *rdh* gene clusters of *D. restrictus* also seems to indicate that the occurrence of specific *rdhA* and *rdhC* genes is not the result of random genetic rearrangements (Supplementary Figure 9). Rather, RdhC proteins have likely co-evolved together with their cognate reductive dehalogenases, suggesting a possible functional relationship. Although the function of RdhC in OHR remains elusive, several sequence features, but also the topology of RdhC in the membrane point toward a possible role in electron transfer, disregarding the initial hypothesis that it is a transcriptional regulator (Smidt et al., 2000). While a transfer of electrons from reduced menaquinones to RdhA enzymes remains disputable in terms of thermodynamics, one could conceive that a flavoprotein like RdhC may render this reaction feasible by a mechanism of electron bifurcation.

In this study, we have unambiguously shown that, in its native state, PceC displays a covalently-bound FMN cofactor and that the FBD of PceC is oriented toward the exocytoplasmic face of the membrane, similarly to the topology of NosR from *Pseudomonas stutzeri* (Wunsch and Zumft, 2005). As for threonine-166 of RnfG from *Methanosarcina acetivorans* (Suharti et al., 2014), site-directed mutagenesis of rFBD clearly confirmed that threonine-168 of PceC is the covalent FMN-binding site. Moreover, reconstitution of the FMN-binding site had a significantly positive effect on folding and solubility of the rFBD protein *in vitro*. The topology of the FBD of PceC, and more generally RdhC, invites to consider the possibility that RdhC proteins may play a role in electron transfer toward the reductive dehalogenase. This hypothesis is in line with the abolition of the nitrous oxide reductase activity of NosZ in presence of a NosR variant, for which the FBD was deleted (Wunsch and Zumft, 2005). Moreover, the midpoint redox potential of flavoproteins ranges from +153 mV to as low as -495 mV (Fagan and Palfey, 2010), a value that is highly variable in comparison to free FMN (-205 mV, Draper and Ingraham, 1968). The redox activity of rFBD and full-length PceC needs to be characterized and will be the focus of further investigation.

In addition to the N-terminal FBD, RdhC sequences share a common membrane module (four membrane segments and two CX_3_CP motifs) with NapH and NosR. The striking difference between RdhC and these proteins is the lack in RdhC of the two 4Fe–4S binding motifs in the C-terminal part. Nevertheless, NapH of *Wolinella succinogenes* was shown to participate with NapF and NapG in electron transfer to the periplasmic nitrate reductase. The membrane segments of NapH were proposed to act as the quinol-oxidizing domain by receiving electrons from the quinol pool and donating them further to redox centers of NapG (Kern and Simon, 2009a). A similar role is conceivable for the membrane domain of RdhC proteins. The mutation of the CX_3_CP motifs in NapH has further demonstrated that both motifs had a severe effect on the integrity of NapH as its partner protein NapG was not associated with the membrane (Kern and Simon, 2008). Moreover, the first cysteine motif of NapH is required for the interaction with NapF and for electron transfer during nitrate respiration (Kern and Simon, 2009b). Similarly, variants of the first cysteine motifs in NosR from *P. stutzeri* completely abolished the activity of NosZ (Wunsch and Zumft, 2005). Whether the CX_3_CP motifs of RdhC build disulphide bridges, like in thioredoxins, or a 2Fe–2S center is not known. However, the former scenario seems to be more likely, since not more than eight atoms of Fe have been detected per molecule of NosR, corresponding to the two 4Fe–4S centers (Wunsch and Zumft, 2005). The high degree of sequence conservation of the CX_3_CP motifs in RdhC proteins and the analogy to NapH and NosR suggest that these motifs are likely determinant for the function of RdhC and will require further investigation.

*In vitro* reconstitution of rFBD protein was made possible with the use of recombinant Ftp1. Our results with Ftp proteins of *D. hafniense* add up to the functional reassignment of ApbE proteins from the role in thiamine synthesis (Beck and Downs, 1998) to a flavin-binding protein (Boyd et al., 2011) and recently to flavin-trafficking proteins (Deka et al., 2015). In contrast to *D. hafniense* TCE1 where both *ftp* genes are encoded in an operon with unknown function, the *ftp* (*apbE*) gene in the genome of *D. restrictus* is located within the operon coding for the Twin-arginine translocation system in a region of the genome harboring 10 *rdh* gene clusters (Rupakula et al., 2013). This suggests a dedicated role of Ftp toward the assembly of RdhC proteins in *D. restrictus*. This is supported by the results of the identification of possible protein candidates with covalently-bound FMN in the theoretical proteomes of *D. hafniense* TCE1 and *D. restrictus* PER-K23. The FMN-binding motif [DN]X_2_[ST]G[AS]TX[ST], as defined previously (Deka et al., 2015), was identified in 13 and 11 proteins in *D. hafniense* and *D. restrictus*, respectively. While all proteins but one are RdhC homologs in the latter, the functional diversity of FMN-binding proteins is much higher in *D. hafniense* (data not shown).

## Conclusion

**FIGURE 8:**
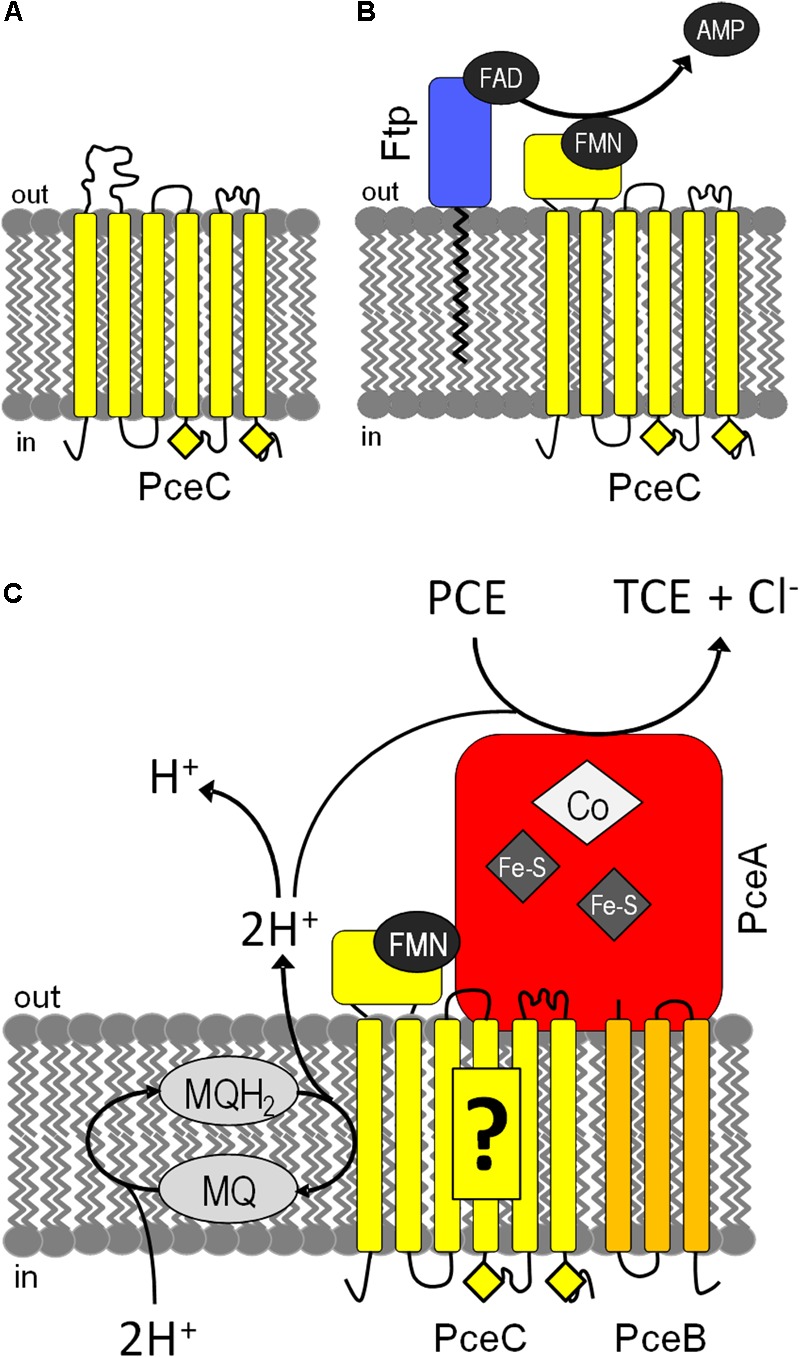
Tentative models for the assembly and function of PceC in *D. hafniense*. **(A)** PceC (in yellow) is likely inserted in the cytoplasmic membrane while the FBD is in an unfolded state. **(B)** On the exocytoplasmic face of the membrane, Ftp (in blue) catalyzes the hydrolysis of FAD to FMN and AMP, and attaches FMN to the threonine-168 of PceC, resulting in the folding of the FBD. **(C)** PceC is hypothesized to build a protein complex with PceA (in red) and PceB (in orange), and to play the role of electron transfer from the menaquinol (MQ) pool to the redox centers of PceA via the FMN cofactor. Yellow diamonds represent the conserved CX_3_CP sequence motifs.

A tentative model for the assembly and function of PceC in *D. hafniense* is presented (**Figure 8**). It is speculated that PceC is targeted to the cytoplasmic membrane in a partially unfolded state via either the signal recognition particle (SRP) or the secretion (Sec) pathway (Luirink et al., 2012) before the FMN cofactor is inserted. Then, the lipid-anchored Ftp catalyzes the hydrolysis of FAD and transfers FMN into the FBD of PceC (**Figure 8A**). The membrane-bound redox PceC protein is proposed to build a complex together with the reductive dehalogenase PceA and with PceB, the predicted membrane anchor of PceA. The membrane segments of PceC would receive electrons from the menaquinol pool and reduce the FMN cofactor, which in turn transfers the electrons to PceA (**Figure 8B**). The mechanism of electron transfer through the redox centers and the implication of the conserved cysteine motifs in the electron transfer remain unknown. These questions need to be addressed in order to challenge the hypothesis of the involvement of PceC in the thermodynamically unfavorable electron transfer from reduced menaquinones to the redox centers of PceA.

## Author Contributions

GB and RH: conducted the experiments and participated in writing the manuscript. MW and AR: conducted the experiments. JM: conceived and led the project, conducted the experiments, and wrote the manuscript.

## Conflict of Interest Statement

The authors declare that the research was conducted in the absence of any commercial or financial relationships that could be construed as a potential conflict of interest.

## Footnotes

1 http://www.sacs.ucsf.edu/TOPO2/

2 https://genome.jgi.doe.gov/
