# Supplementary material for: The Membrane-Bound C Subunit of Reductive Dehalogenases: Topology Analysis and Reconstitution of the FMN-Binding Domain of PceC
Source: Front Microbiol. 2018 Apr 24;9:755. doi: 10.3389/fmicb.2018.00755 (PMC5928378; doi:10.3389/fmicb.2018.00755)
Supplement: Supplementary file 5 [file Presentation_1.pdf]

Supplementary Material

The membrane-bound C subunit of reductive dehalogenases: topology analysis and reconstitution of the FMN-binding domain of PceC

Géraldine F. Buttet, Mathilde S. Willemin, Romain Hamelin, Aamani Rupakula and Julien Maillard*

*** Correspondence:** julien.maillard@epfl.ch

1 Supplementary Data

1.1 Sequence analysis of RdhC proteins

PceC from *Desulfitobacterium hafniense* TCE1 (CDX02972.1) was initially used as seed in BlastP analysis (NCBI website, (Altschul et al., 1990)) with an expect threshold value of e^{-14}. After removing short and extended sequences, a selection of 300 sequences was aligned and the corresponding tree as drawn with iTOL (Letunic and Bork, 2016). Eight clusters were considered, and one sequence of each cluster was used as seed in new independent BlastP analyses. All sequences with expect threshold below e^{-10} were collected (730 non-redundant sequences), among which 484 sequences with length between 322-521 amino acids (length of PceC ± 100 amino acids) were considered. Manual curation of the selection was made by removing sequences showing a distinct homology to NosR homologs, sequences lacking any of the conserved FMN-binding site or cysteine motifs and truncated sequences, which resulted in a final selection of 433 sequences. Clustering analysis with sequence identity threshold at 95% delivered a selection of 236 sequence clusters. Last, a more conservative selection was made by removing all orphan RdhC sequences and sequences found in bacterial genomes that did not code for any RdhA homolog. This resulted in a final selection of 117 sequence clusters, each of them displaying between 1 to 9 unique sequences (**Supplementary Table 2**). Cluster heads were arbitrarily chosen, aligned and displayed in a sequence likelihood tree (**Figure 2**).

1.2 Mass spectrometry analyses of the reconstituted rFBD protein

1.2.1 Mass spectrometry analysis of the intact rFBD protein

Q-TOF MS analysis of the intact reconstituted rFBD protein was done by L. Menin (ISIC, EPFL, Lausanne) as described before (Prat et al., 2012).

1.2.2 Top-down mass spectrometry of the rFBD protein

To confirm the exact localisation of the FMN on the T168 of the PceC protein (T129 of the recombinant 19 kDa rFBD protein), the following workflow was implemented on Orbitrap FTMS platforms (L. Menin, ISIC, EPFL, Lausanne).

1.2.2.1 Experimental part

Experiments were carried out using Orbitrap QExactive HF interfaced with a Dionex UPLC-Ultimate-3000 system. LC separation was performed with a C4-column (Waters), 1 x 150 mm, using a gradient of A (H_2O-HCOOH 0.1%) and B (CH_3CN-HCOOH 0.1%) as mobile phases. First a full MS scan run was acquired using a high-resolution scan of 140 K. Then HCD fragmentation was performed using a mass selection window of 200 Th centered on the m/z 915.8 ion (charge state +21) with a collision energy of 10 eV. A total of 10 consecutive LC experiments were acquired for the rFBD protein sample. In parallel with a standard .raw file containing only mass spectra, the time-domain signals (transients) were acquired using a commercial built-in high-performance data acquisition systems, the FMS Booster X1, developed by SpectroSwiss (EPFL Innovation Park, Switzerland). The spectra/transients were summed across all LC-MS/MS runs, processed with absorption mode FT, the mass spectra are re-calibrated and baseline corrected to generate an accurate peak list (using Peak-by-Peak software) in a mzxml format. High resolution HCD mass spectra of the protein (19 kDa) were analysed with MASH-Suite Pro and Prosight Lite v1.3 software. In parallel, the in-house developed free-access ChemInfo.org algorithms was used for predicting and matching the experimental tandem spectra to theoretical fragment ions, in particular internal fragment ions. For processing, up to 9 protons was allowed, 3 groups ($C_{17}H_{21}N_4O_9P$, $C_{17}H_{19}N_4O_8P$ or nothing) tested with a comparison zone of -1.5 to 6.5 around the monoisotopic theoretical peak. The minimum similarity allowed was set to 85%.

1.2.2.2 Results

The MS/MS spectra generated after multiplexing and advanced FTMS data analysis framework was deconvoluted using MASH Suite Pro and the ions mass list was imported in ProSight Lite. The FMN group ($C_{17}H_{19}N_4O_8P$) was placed on different Threonine positions, looking at the number of b and y fragment ions assigned. The Graphical fragment map showing the best results, i.e. the position of FMN leading to the highest number of fragment ions assigned and identified for the rFBD protein, is displayed on **Supplementary Figure 8A**. A total of 37 b-ion fragment could be assigned with an average mass error of 1 ppm but none of them contained the FMN group (**Supplementary Table 3**). Regarding the C-terminal end of the protein, 32 y-ions were assigned (**Supplementary Table 4**) with an average mass tolerance of 1.1 ppm, among them the first 21 y-ions do not have the FMN adduct. The last unmodified y-ions is y39, whereas the first FMN-Y ion is y43. This result demonstrated that the FMN adduct is most probably located in the string of the 4 residues $GST_{129}V$ in the protein.

In order to confirm the FMN localisation on the T_{129} of the protein, the in-house ms-cheminfo.org algorithm was used to assign internal fragments and detect FMN-internal fragments in the region of

the threonine. None of the commercial software allow assignment of MS/MS internal fragments in an intact protein.

A total of 33 FMN-internal fragment ions could be assigned with a similarity score > 85%. **Supplementary Figure 8B** shows the graphical fragment map restricted to internal fragment ions bearing the FMN ($C_{17}H_{19}N_4O_9P$) group and identified with a similarity score > 85%. The majority of FMN-internal fragments found is located in the same region (region II) and include T_{129}. The other region (I) located in the N-terminal part of the protein has only 3 fragment ions found with good similarity scores. Because a threonine is not present in the sequence involved and because no FMN-b ions could be detected in the N-terminal part of the protein, we conclude that the binding site region of FMN is certainly in region II. The smallest FMN-internal fragment ion found are b137y44 and b134y44, with high similarity scores between experimental data and theoretical, respectively 96% and 5%. The first modified Y-ion identified was the FMN-y44 fragment, assigned with a similarity score of 96% (**Supplementary Figure 8C**).

In conclusion, the localisation of the FMN-binding site on the rFBD protein was restricted to a string of the 4 residues $GST_{129}V$ in the protein, with the threonine T_{129} being presumably the binding site.

1.3 Reconstitution by dilution

FMN assembly in rFBD protein was done initially by 100-fold dilution of urea-denatured rFBD (100 μL of P1) in 10 mL reconstitution buffer (50 mM Tris-HCl, pH 7.5, 100 mM NaCl, 5 mM $MgSO_4$) supplemented with 0.1 mM FAD and 0.18 mg of rFtp1-containing *E. coli* protein extract. A control reaction without rFtp1 was included. Reaction mixtures were incubated for 45 min at room temperature. The samples were concentrated to 0.5 mL with a centrifugal filter (Amicon, Merck-Millipore, Schaffausen, Switzerland). Ten μL aliquots were run on SDS-PAGE following standard protocol (Sambrook et al., 1989), then FMN-containing proteins were detected under UV-visible illumination. Last, the gel was stained with Coomassie Blue (0.1% (w/v) Coomassie Blue R250 in 10% (v/v) acetic acid and 40% (v/v) ethanol).

2 Supplementary Figures and Tables

2.1 Supplementary Figures

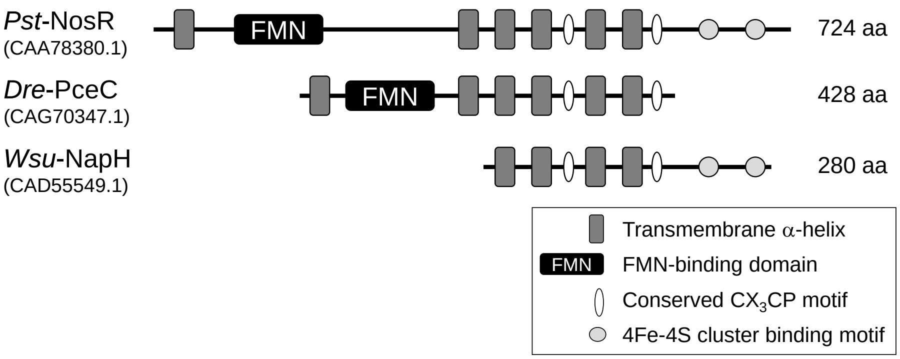

Supplementary Figure 1. Protein domain comparison between PceC (*Dehalobacter restrictus*), NosR (*Pseudomonas stutzeri*) and NapH (*Wolinella succinogenes*). Please note that, based on sequence alignment with closely related sequences, PceC sequence from the original database entry (CAG70347.1) has been manually corrected to include the N-terminal transmembrane α-helix. Evidence for the presence of this helix was obtained by proteomic analysis.

A

```
  1 MKIKKNKAELENRRGWEYYYQFSLLITAIIAILYGVFWAPQSVDYKGIIQ
 51 KNVLGVISIEKMMGNQHAYKIDTAQGRFYAVCDSAIGYQSKVEAMTIVNE
101 KGLIEKVIITKQGETPVFFERLTDQKYFDGFQGLAIKEPIYLGGAYGYSG
151 YLGSIKTNNYIDTVTGSTVSSHAVAEAVNKGNSYLSGQFFNTQWANPYDL
201 FQLSWKDMAMIAMFLIAFASAFIKKLVKIRLAFLLVSVVVLGFLVNQFVT
251 GSLLLSAITLQIPRTTNLKWYVLMAGSLGFIILLGKNLYCAWICPFGAVQ
301 EIINKAAGFKSLNISQKTIKIIRLVAPTIILWALLLGTLLGDYGTLDYQP
351 FGALFLFKSVWLMWLMLPIFLFMSLFISRFYCKFFCPVGFIFNLLNRWRN
401 EEVRIWKQRVDRLKRKKKEKQETLSSHS
```

B

```
  1 MGQSVDYKGIIQKNVLGVISIEKMMGNQHAYKIDTAQGRFYAVCDSAIGY
 51 QSKVEAMTIVNEKGLIEKVIITKQGETPVFFERLTDQKYFDGFQGLAIKE
101 PIYLGGAYGYSGYLGSIKTNNYIDTVTGSTVSSHAVAEAVNKGNSYLSGQ
151 FFNTQWANPYDLLEHHHHHH
```

Supplementary Figure 2. Sequence analysis of PceC from *D. hafniense* TCE1. (A) The protein sequence of PceC is given with the predicted transmembrane α-helices (underlined), the FMN-binding domain (highlight in yellow) with the conserved threonine residue (in green) and the two conserved CX_3CP motifs (in red). (B) Sequence of the recombinant FMN-binding domain of PceC as produced. The conserved threonine residue is indicated in green. Additional residues at the N- and C-termini are indicated in blue.

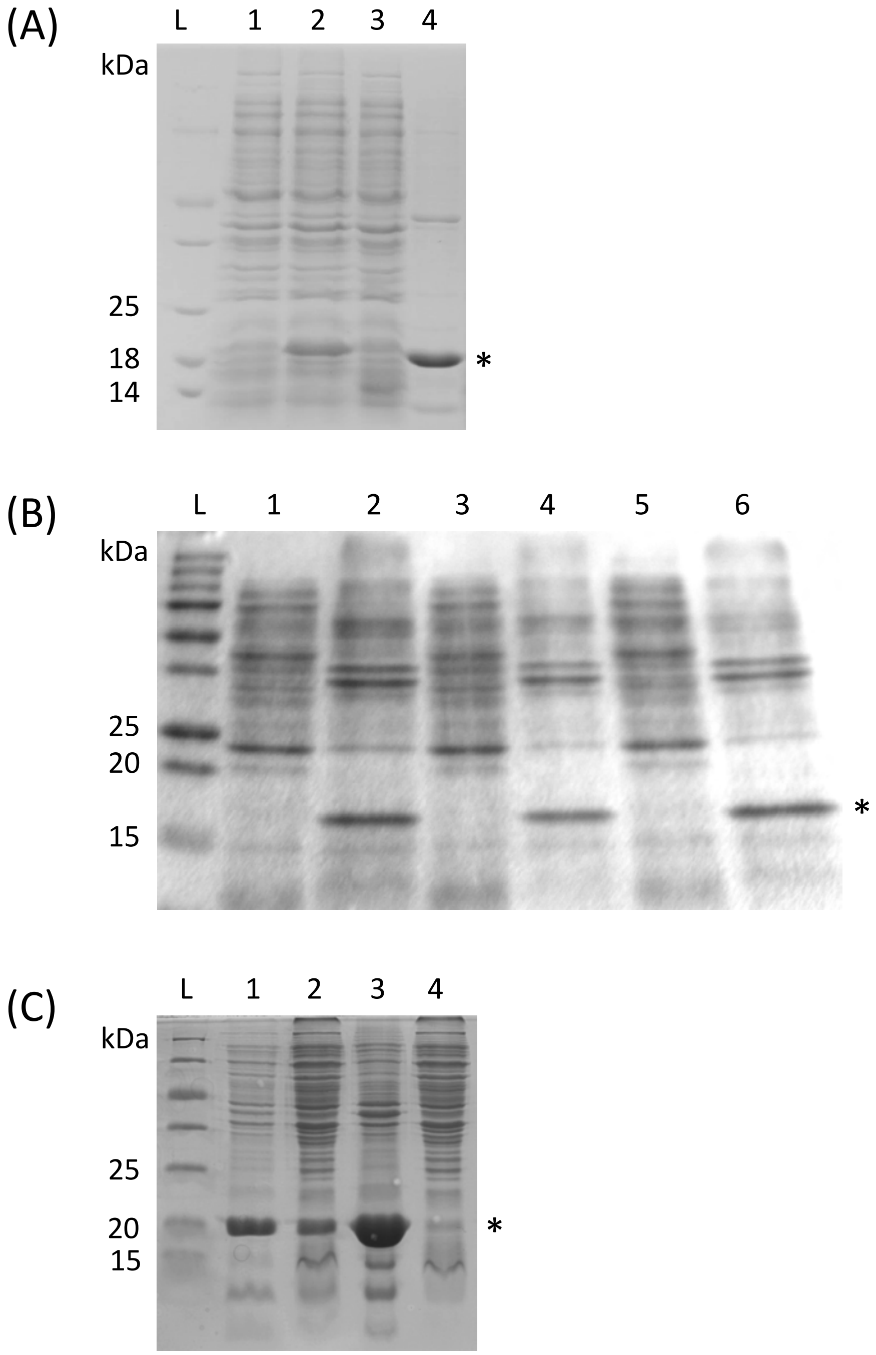

Supplementary Figure 3. (A) Heterologous production of rFBD and fractionation of *E. coli* biomass. Lanes: L, protein ladder; 1, non-induced cells; 2, cells induced with IPTG; 3, soluble fraction; 4, insoluble fraction. (B) *In vivo* rFBD production in presence of flavins (FMN: lanes 1,2; riboflavin: lanes 3,4). Biomass samples were fractionated into soluble and insoluble fractions. Lanes: L, protein ladder; 1,3,5, soluble fractions; 2,4,6, insoluble fractions. (C) Large-scale production of rFBD inclusion bodies by auto-induction. Lanes: L, protein ladder; 1, auto-induced cells; 2, total cell extract; 3, insoluble fraction; 4, soluble fraction. The rFBD protein is indicated by asterisks in all three panels.

(A)

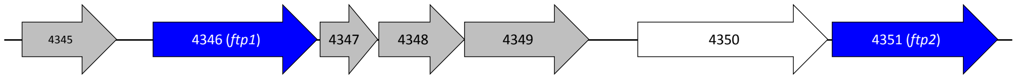

(B)

```
                                                   1
DeshaDRAFT_4346      1 MFDKKKCGRKKLSFVAVALITLGL-LTACNGKPVQQEE--VKKFESTDIAMGTVISQRVF
DeshaDRAFT_4351      1 MQNLRPSSFKSRLLVMLICVCLSFSLVGCLSAETKVKE--FEPVVETTFLMGTVAKITIY
Kpn_KPK_2905         1 -----------------------------------MSD--NRVYSYSAVLMGSPILLKLY
Kpn_KPK_1517         1 ---------MDMTFFRAALLGACVLLSGCDSATTPASP-ASTATVLDGKTMGTFWRVSVI
Eco_PRK10461         1 ---------MEISFTRVALLAAALFFVGCDQKPQPAKTHATEVTVLEGKTMGTFWRASIP

                                                2
DeshaDRAFT_4346     58 GDN-GQAAIDAALEKIKSLEALLTFN--APGGDVNKLNDYAGKQSVELQPETLLVLKESQ
DeshaDRAFT_4351     59 DEIKDNEIFQRVFDRLTDIEQRMTINDDYPNSEIIQLNKASGKEFVKINPDTFYVLEKAK
Kpn_KPK_2905        24 SHD--EALASRVFQLIKRYEDLLTVN--RAESQVMDINHAAGRHPVTVSRPVFQLIQCAK
Kpn_KPK_1517        51 GVDEAKAEALRAKVQAQLDADDRLLSTWKNDSALMRFNHAADTRPWPVSEAMVDIVTLSL
Eco_PRK10461        52 GIDAKRSAELKEKIQTQLDADDQLLSTYKKDSALMRFNDSQSLSPWPVSEAMADIVTTSL

DeshaDRAFT_4346    115 EVAELSGGAFDVTVGPIVKSWGIGTDN--ARIPSETELKELLPLVNYKNLLIEGN--TAY
DeshaDRAFT_4351    119 YLAELSQGKFDITVGPIVKLWNIGSDN--ARVPGIDEINKKLPLVDYHNLILDKDNSSAK
Kpn_KPK_2905        80 AASMVRDSAFNLAIGPLVKLWRIGFHG--HSVPDAADIRARLALTRPQEVILDETTCSVF
Kpn_KPK_1517       111 RIGAKTHGAMDITVGPLVNLWGFGPDKQPVTTPDAQAIAAAKARTGLQHLQVINQSGRQF
Eco_PRK10461       112 RIGAKTDGAMDITVGPLVNLWGFGPEQQPVQIPSQEQIDAMKAKTGLQHLTVINQSHQQY

                                       3
DeshaDRAFT_4346    171 LKQ--AGQMVDLGGIAKGYAGDAAIEVYKKQGITSAFINLGGNVVTLGTKPDGSSWTVGV
DeshaDRAFT_4351    177 LNQ--EGMVVDLGAIAKGYAADEAVKILKEAGIEHAIVNLGGNIVAMNTKLDGSLWRLGL
Kpn_KPK_2905       138 LQQ--PGMELDLGAIAKGYIADRVRDYLRQQVEKALINLGGNVHTLG------EWTIGL
Kpn_KPK_1517       171 LQKDIPDLFVDLSTVGEGYAADHLARLMEQEGISRYLVSVGGALVSRGMNGEGKPWRVAI
Eco_PRK10461       172 LQKDLPDLYVDLSTVGEGYAADHLARLMEQEGISRYLVSVGGALNSRGMNGEGLPWRVAI

                                                3   3
DeshaDRAFT_4346    229 RNPRPAGEEDQIVGMITVADKAVVTAGDDQRYFEVDGVRYHHILNPHTGYPAQSDLMSVT
DeshaDRAFT_4351    235 QDPYEIRGKS--MGVVLLNDQTMVSSGTYERYFEEGGKVYHHLIDPDTGYPGENGLISVS
Kpn_KPK_2905       190 KKP--FADAQALIGSLTINGQSVVTSGTYERYFEQDGKRWHHILDPRSGYPLDNELDSVT
Kpn_KPK_1517       231 QKP--TDRENAVQAIVDINGHGISTSGSYRNYYELDGKRISHVIDPQTGQPITHKLVSVT
Eco_PRK10461       232 QKP--TDKENAVQAVVDINGHGISTSGSYRNYYELDGKRLSHVIDPQTGRPIEHNLVSVT

                                3   3
DeshaDRAFT_4346    289 LVTDSSLLADALDTAVYILGLEKGREMLENYGGVEAVFITRDKKIYVTDGLKDS-FEFFD
DeshaDRAFT_4351    293 IITKESINADGLSTGTFLLGLEEGMKMIEKIPDTEAIFITADKKVYVTSGINSSNFEITN
Kpn_KPK_2905       248 VISTDSLDGDIWTTLLFGLGVEKGCAALRQREDIDAIFVTKNRDIILSSPQRLR-FSPLD
Kpn_KPK_1517       289 VIAPTALEADGWDTGLMVLGPEKAQQVVREQGLAVYMIVKEGEGFKTWMSPQFRTFLVGE
Eco_PRK10461       290 VIAPTALEADAWDTGLMVLGPEKAKEVVRREGLAVYMITKEGDSFKTWMSPQFKSFLVSE

DeshaDRAFT_4346    348 ESKEYEFVKD----
DeshaDRAFT_4351    353 PDYHLQTTPSPESK
Kpn_KPK_2905       307 SGYQVIDCTA----
Kpn_KPK_1517       349 KN------------
Eco_PRK10461       350 KN------------
```

Legend

1: Cysteine involved in lipid anchor

2: Asparagine involved in Mg^{2+} binding

3: Residues involved in FAD/metal binding

Supplementary Figure 4. Flavin-trafficking proteins in *D. hafniense* TCE1. (A) Multi-gene cluster harboring the genes encoding both Ftp (loci 4346 and 4351 in blue) and a predicted FMN-binding protein (4350 in white). (B) Sequence alignment of Ftp1 and Ftp2 of *D. hafniense* TCE1 with Ftp of *E. coli* (Eco) and *Klebsiella pneumoniae* (Kpn). Conserved residues are indicated with number.

(A)

```
seq  MLQSARLVTI SNKEGLEIKE LELLFDKKKC GRKKLSFVAV ALITLGLLTA 50
pred IIIIIIIIII IIIIIIIIII IIIIiiiiii iiiiHHHHHH HHHHHHHHHH

seq  CNGKPVQQEE VKKFESTDIA MGTVISQRVF GDNGQAAIDA ALEKIKSLEA 100
pred Hooooooooo ooooooOOOO OOOOOOOOOO OOOOOOOOOO OOOOOOOOOO

seq  LLTFNAPGGD VNKLNDYAGK QSVELQPETL LVLKESQEVA ELSGGAFDVT 150
pred OOOOOOOOOO OOOOOOOOOO OOOOOOOOOO OOOOOOOOOO OOOOOOOOOO

seq  VGPIVKSWGI GTDNARIPSE TELKELLPLV NYKNLLIEGN TAYLKQAGQM 200
pred OOOOOOOOOO OOOOOOOOOO OOOOOOOOOO OOOOOOOOOO OOOOOOOOOO

seq  VDLGGIAKGY AGDAAIEVYK KQGITSAFIN LGGNVVTLGT KPDGSSWTVG 250
pred OOOOOOOOOO OOOOOOOOOO OOOOOOOOOO OOOOOOOOOO OOOOOOOOOO

seq  VRNPRPAGEE DQIVGMITVA DKAVVTAGDD QRYFEVDGVR YHHILNPHTG 300
pred OOOOOOOOOO OOOOOOOOOO OOOOOOOOOO OOOOOOOOOO OOOOOOOOOO

seq  YPAQSDLMSV TLVTDSSLLA DALDTAVYIL GLEKGREMLE NYGGVEAVFI 350
pred OOOOOOOOOO OOOOOOOOOO OOOOOOOOOO OOOOOOOOOO OOOOOOOOOO

seq  TRDKKIYVTD GLKDSFEFFD ESKEYEFVKD 380
pred OOOOOOOOOO OOOOOOOOOO OOOOOOOOOO
```

(B)

```
MNGKPVQQEEVKKFESTDIAMGTVISQRVFGDNGQAAIDAALEKIKSLEALLTFNAPGGD
VNKLNDYAGKQSVELQPETLLVLKESQEVAELSGGAFDVTVGPIVKSWGIGTDNARIPSE
TELKELLPLVNYKNLLIEGNTAYLKQAGQMVDLGGIAKGYAGDAAIEVYKKQGITSAFIN
LGGNVVTLGTKPDGSSWTVGVRNPRPAGEEDQIVGMITVADKAVVTAGDDQRYFEVDGVR
YHHILNPHTGYPAQSDLMSVTLVTDSSLLADALDTAVYILGLEKGREMLENYGGVEAVFI
TRDKKIYVTDGLKDSFEFFDESKEYEFVKDLESGKETAAAKFERQHMDSSTSAA
```

Supplementary Figure 5. Sequence of the flavin-trafficking protein (Ftp1) of D. hafniense TCE1. (A) Topology prediction of Ftp1: I, inside; H, transmembrane α-helix; O, outside. The N-terminal lipoprotein signal peptide is underlined. The cysteine residue highlighted in yellow is the predicted lipid attachment point. (B) Sequence features of the recombinant form of Ftp1 (rFtp1) as produced in *E. coli*. The lipoprotein signal peptide was removed. The protein starts with a methionine residue (highlighted in yellow) which replaces the cysteine lipid-binding residue. In green is indicated the C-terminal end addition to Ftp1, including the S•Tag™ (underlined) used for improving the solubility of the recombinant protein.

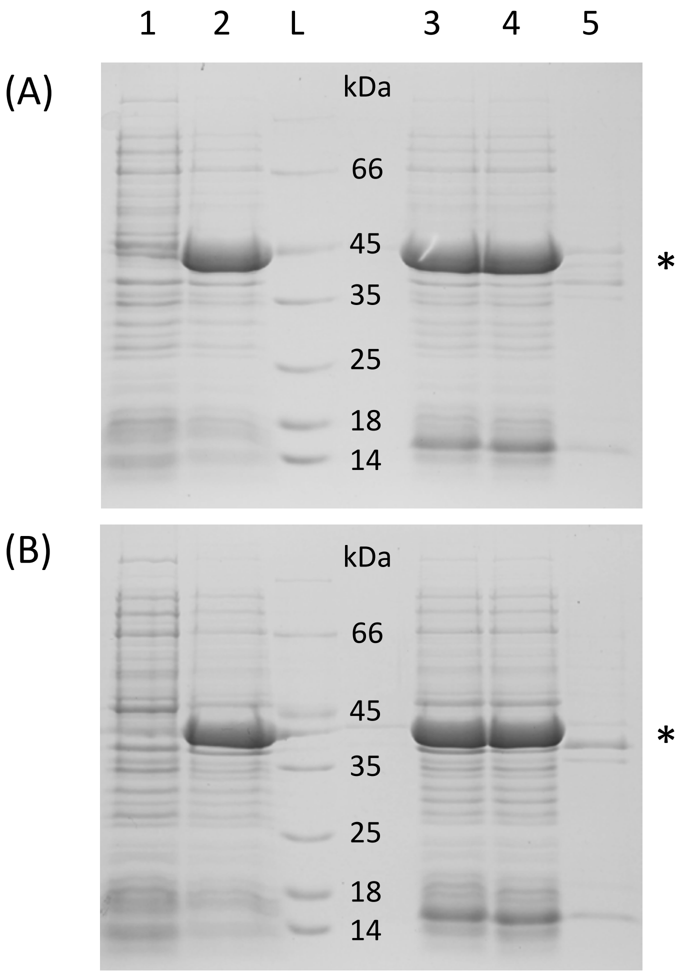

Supplementary Figure 6. Heterologous production of (A) rFtp1 and (B) rFtp2 and fractionation of *E. coli* biomass. Recombinant proteins are indicated with a star. Lanes: L, protein ladder; 1, non-induced cells; 2, cells induced with IPTG; 3, cell-free extract; 4, soluble fraction; 5, insoluble fraction.

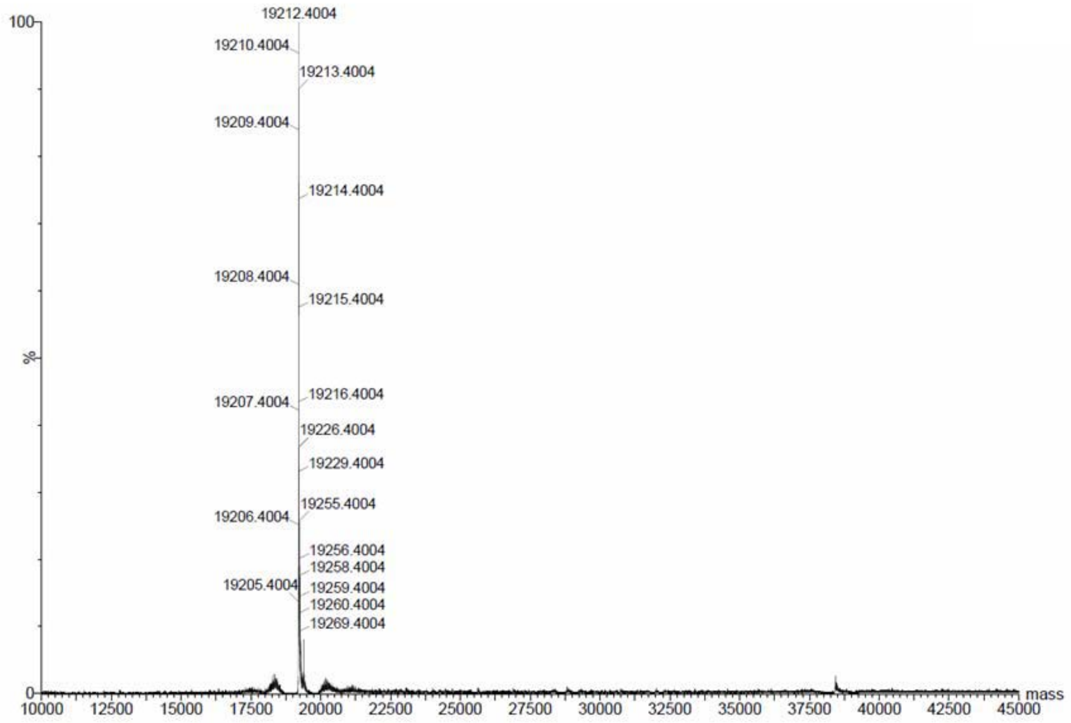

Supplementary Figure 7. Q-TOF MS analysis of the intact reconstituted rFBD protein showing a major peak at 19’212.4 Da, the exact mass of rFBD protein with covalently-bound FMN.

(A)

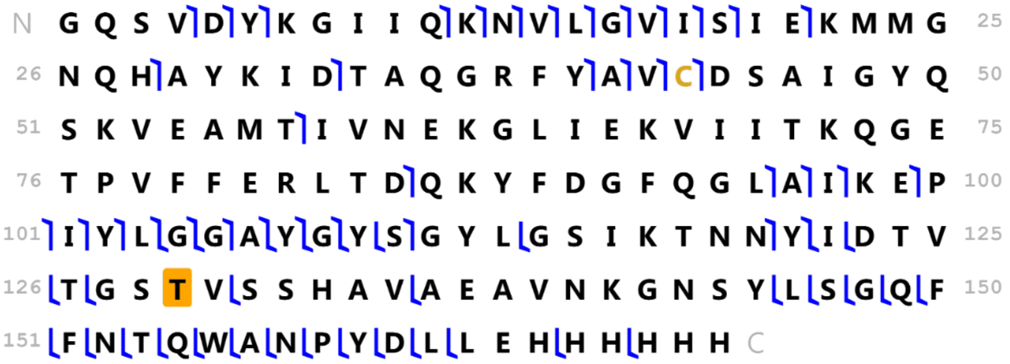

Supplementary Figure 8. (**A**) Graphical fragment map showing results for the rFBD protein after processing with ProSight Lite. The fragment mass tolerance was set to 15 ppm. Please note that the initial methionine residue was cleaved off during protein production in *E. coli*.

(B)

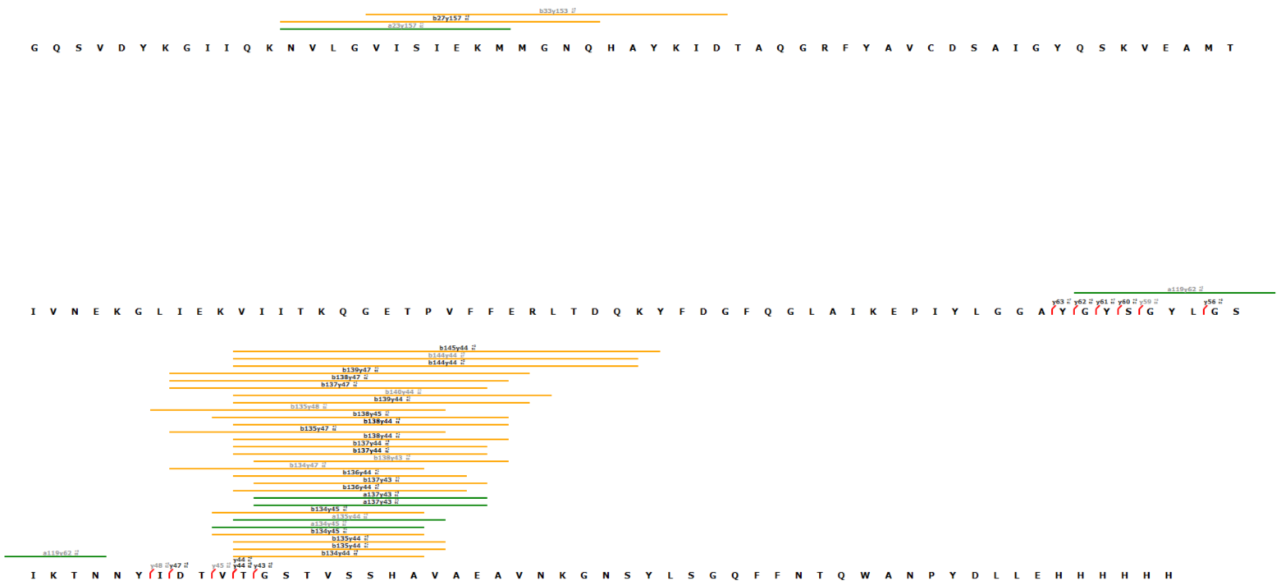

(C)

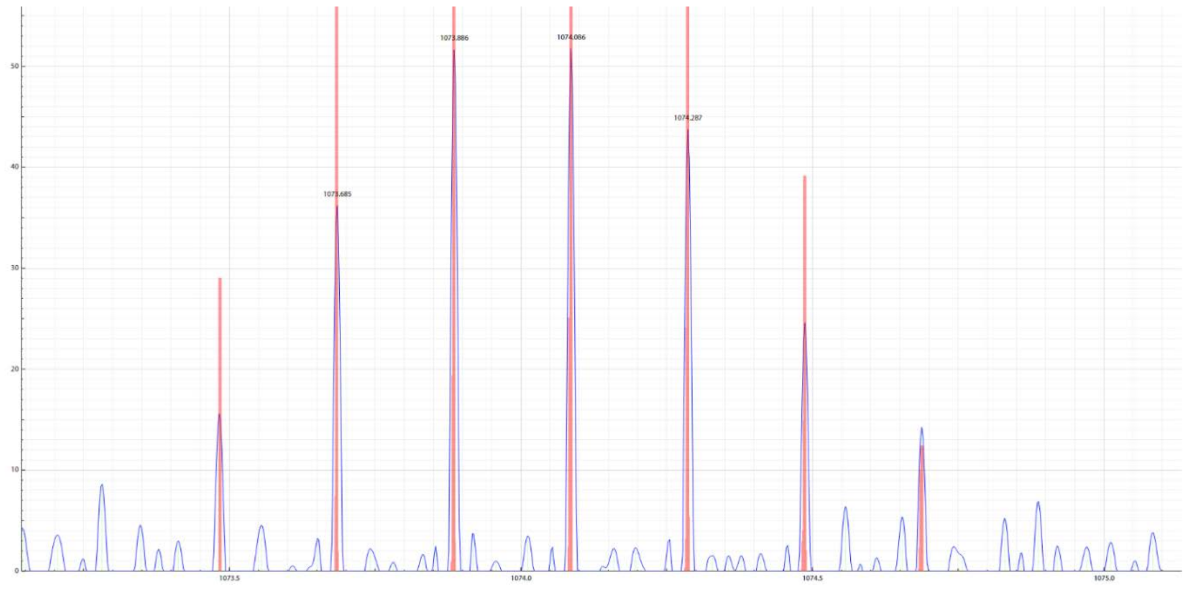

Supplementary Figure 8 (continue). (**B**) Graphical fragment map showing results for the FMN-binding domain of rFBD after processing with ms-cheinfo.org. The graphics only displays internal fragment ions bearing the FMN ($C_{17}H_{19}N_4O_9P$) group, and identified with a similarity score >85%. **(C)** MS/MS spectra of the rFBD protein, focused on the fragment ion at m/z 1073.48 (z=5). The isotopic pattern of the theoretical fragment ion FMN-Y44 (red) matches the experimental spectra (blue) with a similarity score of 96%.

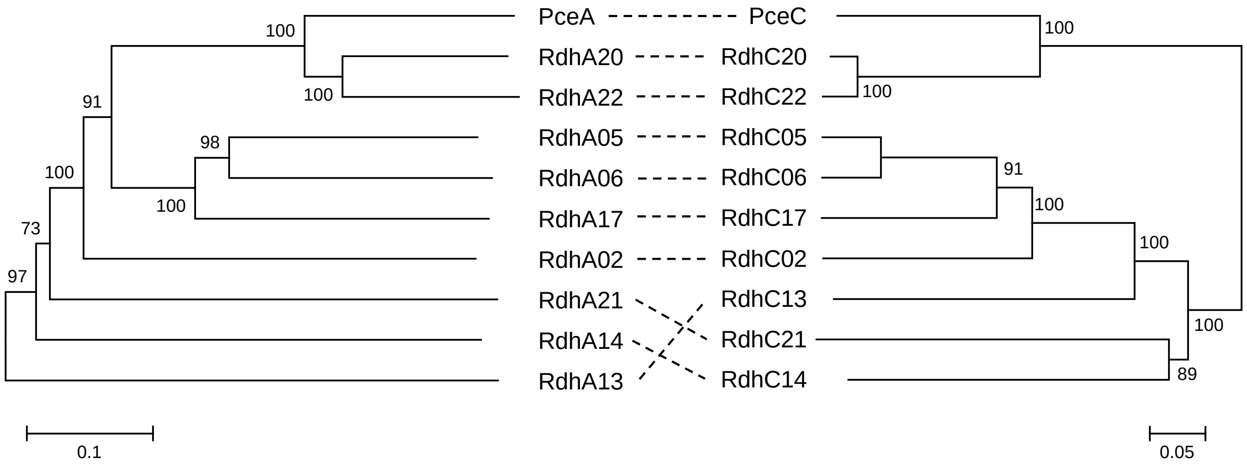

Supplementary Figure 9. Co-evolution analysis of RdhC and their cognate RdhA proteins in *Dehalobacter restrictus*. All RdhC sequences from *D. restrictus* (including PceC) were aligned and plotted with CprA of *D. dehalogenans* as outgroup (not shown), and compared to the tree of their corresponding RdhA sequences (using *D. dehalogenans* CprC as outgroup). Both trees show a significant level of conserved topology, suggesting that RdhC proteins have co-evolved with their cognate RdhA. The numbering of RdhA and RdhC sequences follow the occurrence in the genome sequence and has been described previously (Rupakula et al., 2013).

3 Additional references

Altschul, S.F., Gish, W., Miller, W., Myers, E.W., and Lipman, D.J. (1990). Basic local alignment search tool. *J Mol Biol* 215(3)**,** 403-410.

Letunic, I., and Bork, P. (2016). Interactive tree of life (iTOL) v3: an online tool for the display and annotation of phylogenetic and other trees. *Nucleic Acids Res* 44(W1)**,** W242-245. doi: 10.1093/nar/gkw290.

Prat, L., Maillard, J., Rohrbach-Brandt, E., and Holliger, C. (2012). An unusual tandem-domain rhodanese harbouring two active sites identified in *Desulfitobacterium hafniense*. *FEBS J* 279(15)**,** 2754-2767. doi: 10.1111/j.1742-4658.2012.08660.x.

Rupakula, A., Kruse, T., Boeren, S., Holliger, C., Smidt, H., and Maillard, J. (2013). The restricted metabolism of the obligate organohalide respiring bacterium *Dehalobacter restrictus*: lessons from tiered functional genomics. *Philos Trans R Soc Lond B Biol Sci* 368(1616)**,** 20120325. doi: 10.1098/rstb.2012.0325.

Sambrook, J., Fritsch, E.F., and Maniatis, T. (1989). *Molecular cloning : a laboratory manual.* New York: Cold Spring Harbor Laboratory.
